# Genetic Impacts on the Structure and Mechanics of Cellulose Made by Bacteria

**DOI:** 10.1002/advs.202505075

**Published:** 2025-06-05

**Authors:** Julie M. Laurent, Mathias Steinacher, Anton Kan, Maximilian Ritter, Mario Leutert, Siiri Bienz, David Häberlin, Naresh Kumar, André R. Studart

**Affiliations:** ^1^ Complex Materials Department of Materials ETH Zürich Zürich 8093 Switzerland; ^2^ Wood Materials Science Institute for Building Materials Department of Civil, Environmental and Geomatic Engineering ETH Zürich Zürich 8093 Switzerland; ^3^ WoodTec group Cellulose and Wood Materials Empa – Swiss Federal Laboratories for Materials Science and Technology Dübendorf 8600 Switzerland; ^4^ Institute of Molecular Systems Biology Department of Biology ETH Zürich Zürich 8093 Switzerland; ^5^ Laboratory of Organic Chemistry Department of Chemistry and Applied Biosciences ETH Zürich Zürich 8093 Switzerland; ^6^ NCCR Bio‐inspired Materials ETH Zürich Zürich 8093 Switzerland

**Keywords:** bacterial cellulose, directed evolution, fiber networks, mutations, proteomics

## Abstract

The synthesis of cellulose pellicles by bacteria offers an enticing strategy for the biofabrication of sustainable materials and biomedical devices. To leverage this potential, bacterial strains that overproduce cellulose are identified through directed evolution technology. While cellulose overproduction is linked with a specific genetic mutation, the effect of such mutation on the intracellular protein landscape and on the structure and mechanical properties of the cellulose pellicles is not yet understood. Here, the proteome of bacteria evolved to overproduce cellulose is studied and its effect on the structure and mechanics of the resulting cellulose pellicles is investigated. Proteomic analysis reveals that the protein landscape of the evolved bacteria shows pronounced differences from that of native microorganisms. Thanks to concerted changes in the proteome, the evolved bacteria can generate cellulose pellicles with exquisite structure and improved mechanical properties for applications in textiles, packaging, and medical implants.

## Graphical abstract

A genetic mutation in a protease of a cellulose‐overproducing evolved bacterium significantly affects the proteome, structure, and mechanical properties of bio‐fabricated cellulose pellicles. By overexpressing synthase proteins that build the cellulose‐forming biological machinery of the cell, the evolved bacterium generates dense, stiff, and strong cellulose films for sustainable applications in packaging, textiles, and biomedicine.

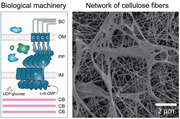

## 1 Introduction

Cellulose‐producing bacteria offer an attractive route for the sustainable fabrication of functional materials for biomedical, textile, and packaging industries.^[^ ^1^, ^2^, ^3^ ^]^ The sustainable nature of this biofabrication process stems from the fact that bacterial cellulose pellicles are produced in water at ambient temperature and can grow using cheap and widely available agriculture waste as carbon source. Because pellicles can grow in vertical farms, the bacterial route significantly reduces the amount of water and land needed for fabrication compared to cellulose‐based materials extracted from wood and agricultural feedstock.^[^ ^4^ ^]^ Moreover, no fertilizers and pesticides are required for bacterial cellulose production. In addition to its intrinsic biocompatibility and biodegradability, bacterial cellulose also features enhanced mechanical properties and high purity without the extensive separation processes required to extract cellulose from plant sources. Simple washing in alkaline solutions leads to cell‐free pellicles that can be directly used after drying or shaped into fibers and films using established spinning and casting technologies. The ability to synthesize and extrude bacterial cellulose fibers into mechanically robust pellicles is provided by a biological machinery that involves a transmembrane protein complex known as bacterial cellulose synthase (Bcs).

**Figure 1:**
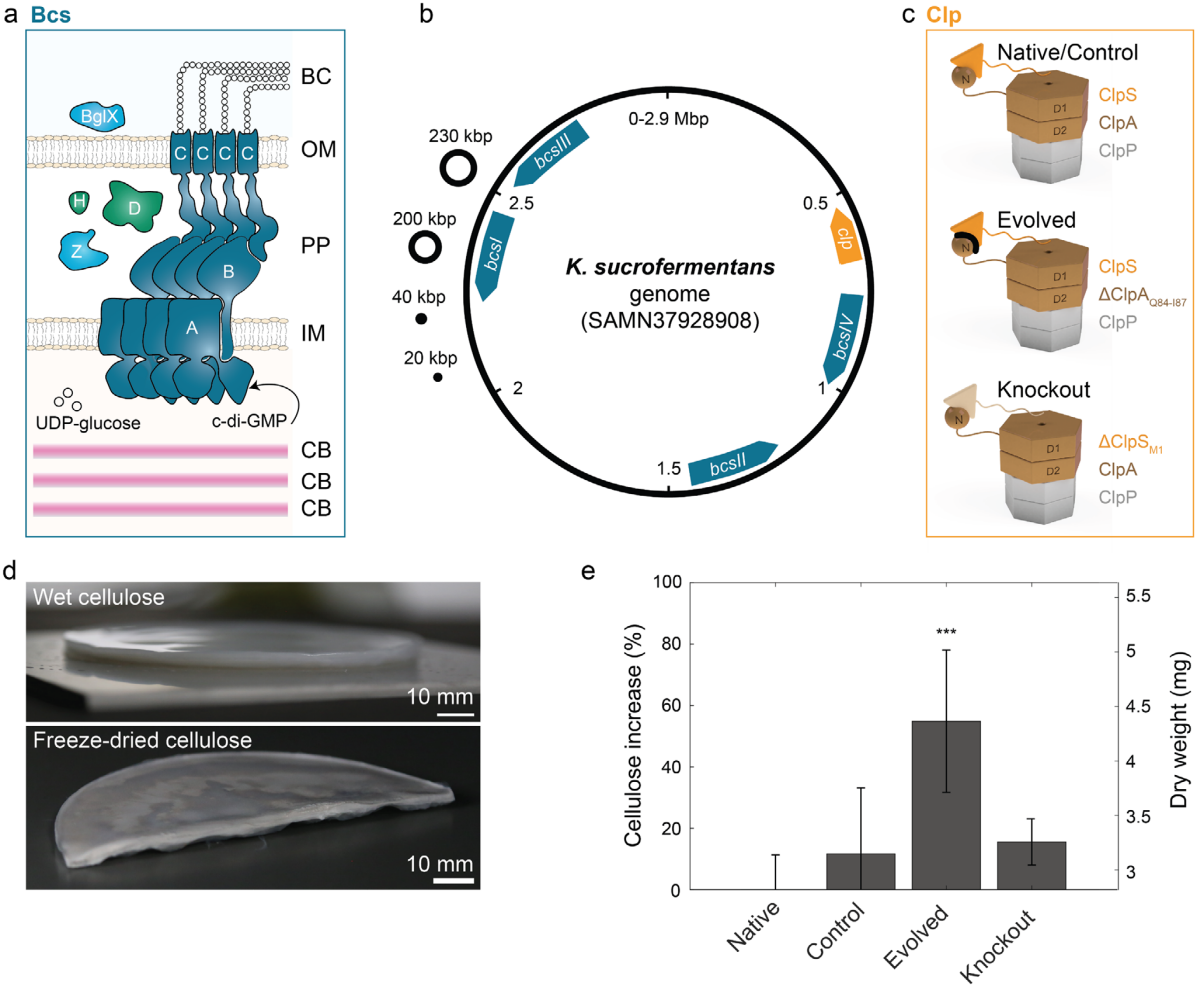
Cellulose‐producing *K. sucrofermentans* bacterial strains: Native, Control, Evolved, and Knockout. a) Schematics of the Bcs protein complex that constitutes the biological machinery responsible for the extrusion of cellulose fibrils. A, B and C represent the protein subunits of the Bcs complex. Proteins involved in hydrolysis (BglX, Z) and crystallization (D and H) of the cellulose chains are also displayed. BC, OM, PP, IM, and CB refer to bacterial cellulose, outer membrane, periplasm, inner membrane, and cortical belt, respectively. Adapted with permission from Abidi *et al.* ^[^ ^5^ ^]^ b) Genome representation of the Native *K. sucrofermentans* strain (SAMN37928908, not to scale), indicating the positions of *bcs* and *clp* operons along the main circular DNA. Adapted from Laurent *et al.*, licensed under CC BY 4.0 (https://creativecommons.org/licenses/by/4.0/)^[^ ^9^ ^]^ c) Schematics of the protease complex ClpAPS, highlighting differences between the investigated strains. Compared to the genetically identical Native and Control bacteria, the Evolved strain displays a 12‐base pair deletion in the gene coding for the protein subunit ClpA (brown), which as a result lacks 4 amino acids at the binding site with ClpS (shown in black). The Knockout strain has a deleted *clpS* start codon, which should lead to the non‐expression of ClpS. Adapted from Kim *et al.*, licensed under CC BY 4.0 (https://creativecommons.org/licenses/by/4.0/)^[^ ^26^ ^]^ d) Photographs of wet and freeze‐dried cellulose pellicles produced by the Evolved strain over an incubation period of 18 days in 100 mL 2x medium. Adapted with permission from Laurent *et al.* ^[^ ^9^ ^]^ e) Cellulose production in 5 mL medium after 12 days of cultivation by the different bacterial strains compared to the Native strain. Statistics: ^***^ *P* < 0.001, *n_pellicles_* = 9. The error bars represent the standard deviations.

The cellulose synthase machinery of bacteria polymerizes glucose and secretes the resulting macromolecule into the extracellular environment using the activated sugar UDP‐glucose as a precursor (**Figure** **1**a).^[^ ^5^ ^]^ In *Komagataeibacter* species, the catalytic core of this secretory protein complex is formed by the subunits BcsA and BcsB, which are anchored in the inner membrane of the cell. These are complemented by the exporter subunit BcsC placed at the outer membrane and by homologs of the cellulases BcsZ and BglX that help in cellulose quality control. Together, these core sub‐units are responsible for the synthesis and secretion of individual cellulose microfibrils that self‐assemble into ribbons after extrusion into the extracellular space. In addition to these molecules, several other proteins have been proposed to take part in the cellulose synthesis and secretion process to enable effective crystallization (BcsD and BcsH) and acetylation (BcsX and BcsY) of the glucan chains. Remarkably, recent research suggests that the self‐assembly of cellulose microfibrils into ribbons is spatially guided by a cytosolic cortical belt, which resembles the cortical microtubules that orient cellulose synthase complexes along the walls of plant cells.^[^ ^5^, ^6^ ^]^ Polymers of BcsD and BcsH have been identified as potential candidates for this cytosolic cortical belt.^[^ ^7^ ^]^

The genes encoding the proteins of the Bcs complex are organized into four operons (*bcsI*, *bcsII*, *bcsIII*, *bcsIV*) in the main circular genome of the bacteria, which consists of 2.9M base pairs and contains most of the genetic information of the microorganism (Figure 1b).^[^ ^8^, ^9^ ^]^ Many of the genes that control or regulate the synthesis of Bcs have been identified and manipulated using synthetic biology tools to enhance the production of bacterial cellulose.^[^ ^2^ ^]^ The synthesis of cellulose by *Komagataeibacter sp*. can be enhanced by manipulating genes to increase the concentration of the UDP‐glucose precursor,^[^ ^10^, ^11^, ^12^, ^13^ ^]^ directly or indirectly overexpress Bcs complex proteins,^[^ ^14^, ^15^, ^16^ ^]^ prevent the competing and unfavorable production of gluconic acid,^[^ ^17^, ^18^, ^19^, ^20^, ^21^ ^]^ or inhibit mutations into non‐cellulose producing variants.^[^ ^22^ ^]^ Despite several reported strategies, synthetic biology approaches often lead to metabolic burdens on cells, which reduce genetic stability and limit industrial up‐scaling.^[^ ^23^ ^]^ This calls for complementary tools to improve bacterial cellulose production.

As an alternative strategy, a high‐throughput microfluidic approach has recently been proposed to direct the evolution of *Komagataeibacter sucrofermentans* toward variants with enhanced cellulose‐producing capability at relatively large scale.^[^ ^9^ ^]^ In this approach, the native bacteria were first subjected to UV‐C irradiation to induce random mutations across the entire genome of the microorganism. The resulting library of mutants was then encapsulated and incubated in nutrient‐rich microscopic droplets with single‐cell occupancy to enable cellulose production in the presence of a specific fluorescent dye. High‐throughput screening of the droplets after incubation allowed for the selection of a cellulose overproducer, referred to as the Evolved strain. Such Evolved strain was able to produce pellicles with 54–70% more cellulose than those fabricated by the Native microorganism. Surprisingly, genetic analysis indicated no mutations in genes encoding the Bcs complex. Instead, the Evolved strain displayed a 12‐base pair deletion in a gene coding for the ClpAPS protease complex. This indicates that the cellulose‐overproducing abilities of the evolved strain are linked to genetic changes, thereby establishing a novel genotype‐phenotype correlation. In spite of these enticing prospects, the possible effect of this mutation on the expression of *bcs* genes and on the properties of cellulose fiber networks are still unknown.

Here, we investigate how the protein landscape in evolved *K. sucrofermentans* affects the structure and mechanical properties of cellulose pellicles produced by this bacterium. First, the genetic and proteomic profiles of the evolved strain are analyzed and compared to those of native, engineered, and control microorganisms. This is followed by a detailed characterization of the chemical composition and crystallinity of cellulose pellicles and fibers using Raman spectroscopy, optical microscopy, and X‐Ray scattering. In these analyses, cell‐free pellicles obtained through a standard washing procedure are compared to as‐grown cellulose samples. Finally, the impact of genetics on the structure and properties of bacterial cellulose pellicles is elucidated by characterizing the fiber network architecture and the mechanical behavior of samples grown from microorganisms with distinct protein abundance profiles.

## 2 Results and Discussion

**Figure 2:**
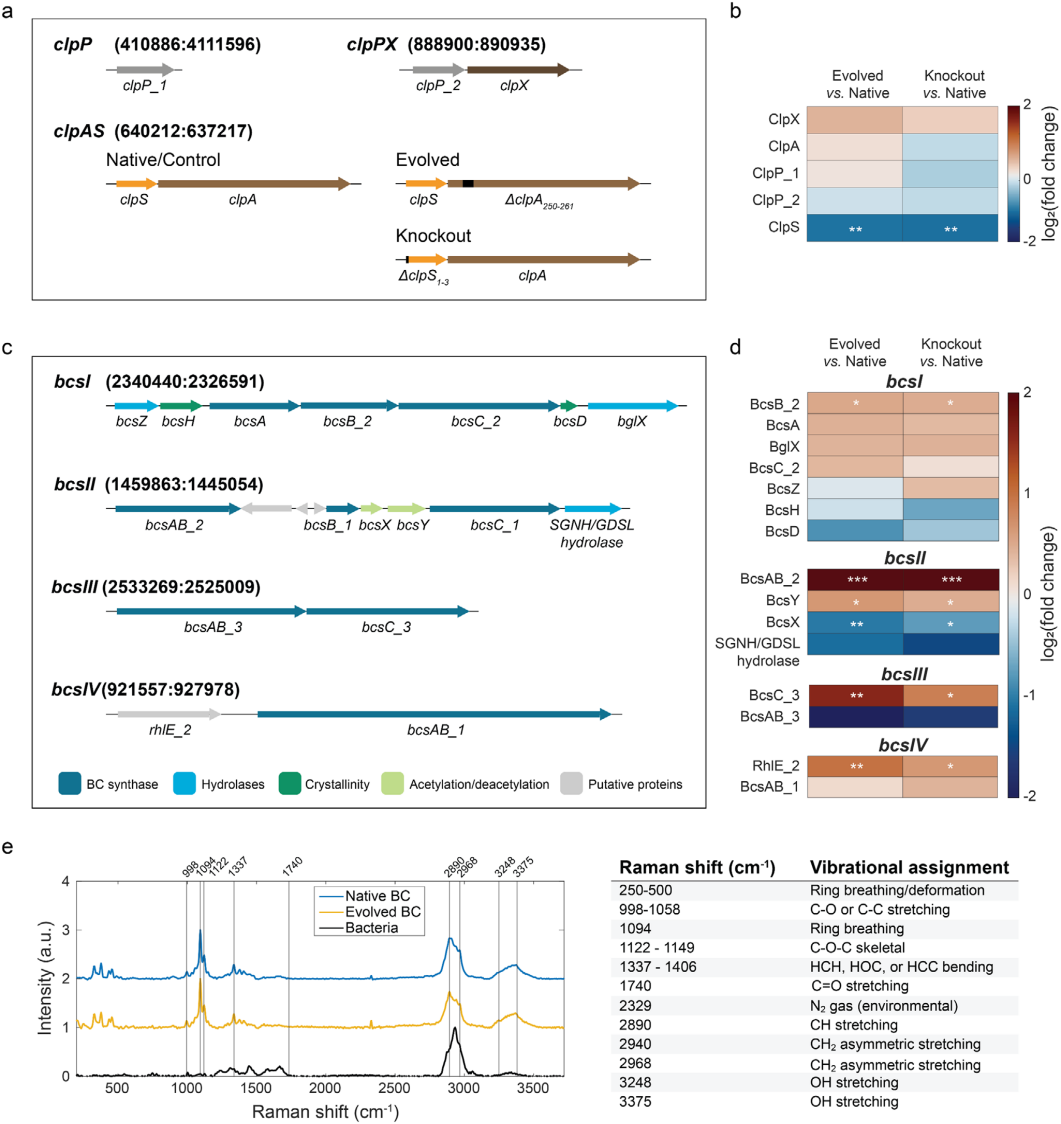
Proteome analysis of the different *K. sucrofermentans* strains and chemistry of the produced cellulose. a) The three *clp* operons that code for the sub‐units of the ClpAPS complex, highlighting the differences in the *clpAS* operon of the different strains. b) Differential protein expression of the ClpAPS complex sub‐units in the Evolved and Knockout strains compared to the Native strain (log_2_ fold change). Dark blue indicates decreased protein abundance and dark red displays increased protein abundance. c) The four *bcs* operons that genetically encode the Bcs complex: *bcsI*, *bcsII*, *bcsIII*, and *bcsIV*. Putative functions of the different genes are color‐coded: *bcs* sub‐units A, B, and C in dark blue; cellulose hydrolases *bcsZ (cmcAX)*, *bglX (bglB_1)*, and SGNH/GDSL hydrolase in light blue; *bcsH (ccpAx)* and *bcsD (acsD)* genes putatively involved in cellulose crystallinity^[^ ^5^, ^7^, ^27^ ^]^ in dark green; *bcsX* and *bcsY (oatA_1)* putatively involved in the deacetylation and acetylation, respectively, of bacterial cellulose^[^ ^3^, ^28^, ^29^ ^]^ in light green; and putative proteins with unknown function in grey. d) Differential protein expression of the Bcs complex sub‐units in the Evolved and Knockout strains compared to the Native strain (log_2_ fold change). Dark blue indicates decreased protein abundance and dark red indicates increased protein abundance in the Evolved or Knockout strains compared to the Native strain. Statistics: ^*^ *P* < 0.05, ^**^ *P* < 0.01, ^***^ *P* < 0.001, *n_samples_* = 3. e) Confocal Raman spectra of freeze‐dried cellulose washed with water produced by the Native strain (blue), the Evolved strain (yellow), and freeze‐dried bacterial cells (black). Characteristic Raman signals of cellulose are listed in the table as well as the expected C═O stretching if acetylation were present. References are provided in Table S2 (Supporting Information).

The role of genetics in the structure and mechanical properties of cellulose pellicles was studied by using three genetically distinct bacterial strains: Native, Evolved, and Knockout. The genomes of these strains show differences in the operons expressing proteins of the ClpAPS protease complex system (Figures 1c and 2a). This protease degrades proteins by unfolding them through ClpA and translocating them into the proteolytic ClpP core for degradation. The protease ability to recognize specific substrates depends on the binding of ClpA to the optional adaptor protein ClpS.^[^ ^24^ ^]^ The Native strain displays the full genome of *K. sucrofermentans*. In contrast, the Evolved and Knockout strains contain genetic modifications that may affect the selective binding between the ClpA and ClpS sub‐units of the protease complex. The genetic modification in the Evolved strain consists of a 12‐base pair deletion in the gene that codes the N‐domain of the ClpA protein, which is the region responsible for selective binding to the ClpS sub‐unit.^[^ ^25^ ^]^ In the case of the Knockout strain, wild‐type ClpA is present, but the bacterium is engineered to prevent the expression of the *clpS* gene, thereby removing any possible binding. To complement the three strains, we also evaluated a Control bacterium that is genetically identical to the Native species but was also exposed to the chemical stresses applied to the Evolved strain during the mutagenesis procedure, but not exposed to UV radiation. Importantly, all investigated strains are equipped with identical copies of the *bcs* operons that are needed for cellulose production.

To quantify the amount of cellulose produced by the different bacterial strains, we measured the dry weight of cell‐free pellicles after cultivation (Figure 1d). The results were combined with our previously reported data^[^ ^9^ ^]^ to showcase the amount of cellulose produced in the pellicles by the distinct bacterial strains (Figure 1e). While the four *bcs* operons remain intact in all evaluated strains, the Evolved bacteria produced macroscopic cellulose pellicles weighing, on average, 55% more than those produced by the Native microorganism. This relative increase in the amount of synthesized cellulose is significantly higher than the 12% and 16% overproduction observed for the Control and Knockout strains, respectively. Because it produces more cellulose after a fixed incubation time, the Evolved strain is also expected to feature a higher pellicle growth rate, thus enabling shorter production cycles during biomanufacturing.

As the mutation in the Evolved strain was not directly found in the *bcs* genes, the possible mechanisms underlying its cellulose overproduction were further investigated by performing proteome analysis of selected strains (**Figure** **2**a–d). First, the analysis focused on the abundance of the ClpAPS complex sub‐units in the genetically different bacteria (Figure 2a,b). The results reveal that the ClpS protein expression is strongly down‐regulated in both Evolved and Knockout strains compared to the Native bacterium. Moreover, the ClpA abundance was not found to be statistically significantly different for all the tested microorganisms. This result confirms the suppression of the *clpS* expression in the Knockout strain. Interestingly, the 12‐base pair deletion in the *clpA* gene of the Evolved strain did not affect the abundance of the ClpA protein; instead, it reduced the expression of ClpS. This decrease in abundance may result from a weaker binding affinity between mutated ClpA and ClpS. Such weaker binding affinity might decrease ClpS expression or decrease ClpS stability, which would lead to the degradation of this protein.^[^ ^24^ ^]^ Because the *clpS* gene is not expected to control cellulose synthesis directly, we hypothesize that the overproduction of fibers by the Evolved strain arises from changes in the abundance of Bcs proteins.

Four *bcs* operons are encoded in the *K. sucrofermentans* genome. The vast majority of genes present in these four operons code for membrane‐associated proteins, BcsABC, that form the bacterial cellulose synthase complex (Figure 2c). Other genes of these operons have been proposed to control the hydrolysis, crystallization, and acetylation of the cellulose molecules synthesized by the bacteria. *bcsI* operon is putatively responsible for the formation of crystalline cellulose through the proteins encoded by the genes *bcsD* and *bcsH*. These are believed to drive the linear organization of *Bcs* complexes during the self‐assembly process of cellulose chains (Figure 1a).^[^ ^5^, ^7^, ^27^ ^]^ *bcsII* is suspected to control the degree of acetylation of the cellulose macromolecules. This would occur via the *bcsX* and *bcsY* genes, which have similar sequences to a deacetylase and an acetyl‐transferase, respectively.^[^ ^3^, ^28^, ^29^ ^]^ Changes in the relative abundance of proteins expressed by these genes might directly affect the amount, chemical composition, and crystallinity of cellulose fibers produced by the microorganism.

Proteome analysis revealed that the Evolved and Knockout strains display a general up‐regulation of the expression of bacterial cellulose synthase complexes in comparison with the Native bacteria (Figure 2d). The expression level of some synthase sub‐units from the *bcsII* and *bcsIII* operons of the Evolved microorganism increased by a factor of 4 and 3, respectively, relative to the Native strain. The greater abundance of Bcs proteins in the Evolved bacteria is expected to be central for the overproduction of cellulose by this variant.

In addition to the direct increase in Bcs expression levels, the Evolved and Knockout strains also showed statistically relevant changes in the abundance of, respectively, 380 and 243 proteins that may indirectly increase cellulose synthesis (Table S1, Supporting Information). Some of the variations are known to enhance cellulose production, while others have not yet been linked to cellulose production or associated processes. For example, the detected increased abundance of OprB porins could enhance the uptake and availability of sugar molecules needed for cellulose production.^[^ ^30^ ^]^ In contrast to this proteomic change, the up‐regulation of a glucose dehydrogenase (Gcd) expression in the Evolved and Knockout strains is a surprising result, since it could suggest an increase in the production of gluconic acid from glucose, thus divering the carbon flux away from cellulose synthesis. This up‐regulation was counterbalanced by a decrease in the abundance of PqqC/D, which is involved in pyrroloquinoline quinone (PQQ) biosynthesis. In the absence of this PQQ co‐factor, Gcd is not able to efficiently convert glucose into gluconic acid.^[^ ^31^ ^]^

Another interesting finding regards the phosphodiesterase (PDE) DosP, which is usually repressed during biofilm formation to increase the concentration of c‐di‐GMP molecules available to activate cellulose synthase complexes.^[^ ^32^, ^33^ ^]^ Considering only the statistically significant data, the proteomic data show that 3 out of 9 encoded DosP are more abundant in the Evolved strain, while only 1 is less abundant (Table S1, Supporting Information). This result suggests that c‐di‐GMP availability does not play a limiting role in cellulose production by the Evolved bacteria. Other changes in the protein landscape that may favor cellulose production by the Evolved microorganism include the up‐regulated expression of proteins involved in glucose synthesis (PpdK and MaeA, Table S1, Supporting Information),^[^ ^34^ ^]^ and acid resistance (AlsD and YdeP, Table S1, Supporting Information). Overall, the proteomic changes detected in the Evolved strain indicate a tightly coordinated metabolic adaptation that optimizes energy production, carbon allocation, and stress resistance, while minimizing resource diversion to secondary functions, culminating in enhanced cellulose production.

Besides the changes discussed above, the proteomic data also showed variations in the abundance of proteins presumed to be associated with cellulose deacetylation and acetylation processes. In the proteomes of the Evolved and Knockout variants, the abundance of putative acetyl‐transferase BcsY was increased, while the abundance of putative deacetylase BcsX was decreased compared to the Native strain. This suggests that the cellulose produced by the Evolved and Knockout variants might feature a higher level of acetylation relative to those synthesized by the Native bacteria. Additionally, despite the statistically small differences, the lower relative abundance of BcsD and BcsH proteins might lead to lower crystallinity in fibers synthesized in the Evolved and Knockout strains.

To investigate the effect of the observed protein imbalance on the acetylation degree of the bacterial cellulose, we performed confocal Raman spectroscopy on pellicles produced by the Native and Evolved bacteria (Figure 2e). In this analysis, the excitation laser was focused on cell‐free regions of freeze‐dried pellicles to assess the chemical composition of the cellulose fibers only. The obtained Raman spectra detected no significant differences in the chemical composition of the cellulose fibers produced by the different strains. The absence of the carbonyl peak associated with acetate groups around 1740 cm^−1^ (C═O) suggests that the cellulose fibers produced by both bacteria are predominantly deacetylated. This indicates that the observed abundance imbalance of BcsX and BcsY in the Evolved strain does not lead to a noticeable impact on the chemical composition of the cellulose pellicles produced by this microorganism.

In addition to chemical composition, the crystallinity of the cellulose synthesized by distinct bacteria was also measured to identify possible effects of the proteome on the molecular order of the polymer at the length scale of fibers and pellicles. Indeed, bacterial cellulose exhibits a hierarchical organization across length scales: the secreted linear β‐1,4‐glucan chains assemble into fibers, which in turn entangle to form macroscale pellicles. To quantify the fraction of crystals in individual cellulose fibers and macroscopic pellicles, we used fluorescence microscopy and Wide‐Angle X‐ray Scattering (WAXS), respectively.

**Figure 3:**
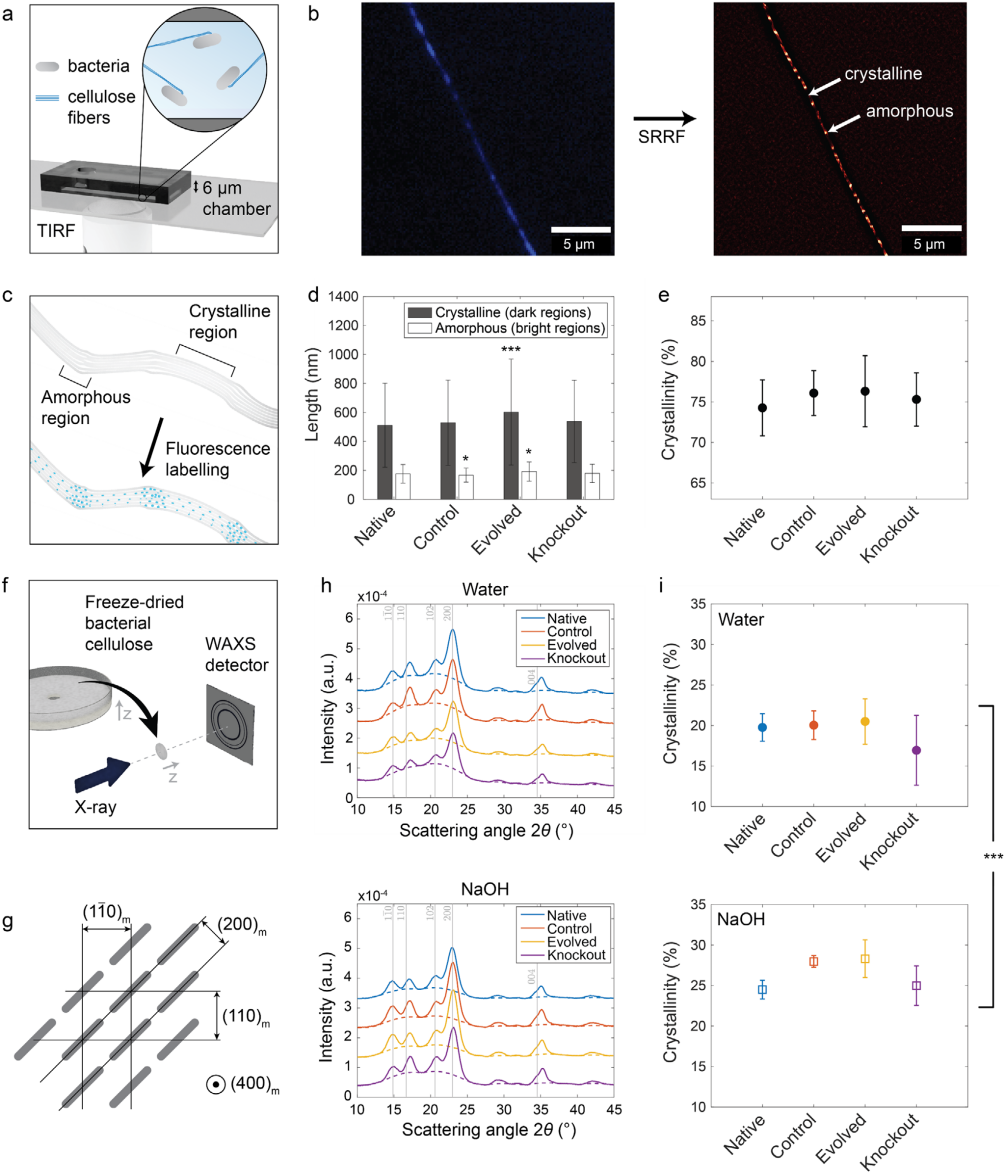
Fiber‐ and pellicle‐level crystallinity of bacterial cellulose produced by the different strains of *K. sucrofermentans*. a) Scheme of the platform used to image single cellulose fibers using Total Internal Reflection Fluorescence (TIRF) microscopy. b) Representative TIRF image of a single cellulose fiber (left) and image processed (right) using Super Resolution Radial Fluctuations (SRRF, ImageJ plugin).^[^ ^35^, ^36^ ^]^ c) Illustration depicting the preferential staining of disordered cellulose regions of the fiber. Bright and dark regions correspond to amorphous (disordered) and crystalline domains, respectively.^[^ ^37^ ^]^ d) Length of dark (black) and bright (white) domains in the cellulose fibers produced by the different strains, measured from image analysis. Statistics: ^*^ *P* < 0.05, ^***^ *P* < 0.001, *n_peaks_* > 380. e) Crystallinity of bacterial cellulose fibers produced by the different strains, considering the length of the dark domains over the length of all domains. No statistically significant differences were found between the strains (*n_fibers_* ≥ 22). f) Scheme of wide‐angle X‐ray scattering (WAXS) setup used to analyze freeze‐dried bacterial cellulose samples. g) Schematic of the lattice planes in cellulose Iβ (monoclinic unit cell). h) Representative WAXS diffractograms of bacterial cellulose obtained from the different bacterial strains, either washed with water (top) or NaOH (bottom). The dashed lines show the fitted backgrounds. The expected cellulose Iβ peak positions are annotated in grey (database: 00‐056‐1718, PDF‐5).^[^ ^50^ ^]^ The diffractograms were vertically shifted for aided readability. i) Crystallinity of the different bacterial cellulose pellicles, calculated from the area under the peaks and subtracting the fitted amorphous background. No statistical difference was observed between the cellulose produced by the different strains, but the samples treated with NaOH showed a statistically significant increase in crystallinity compared to samples treated with water. Statistics: ^***^ *P* < 0.001, *n_pellicles_* ≥ 3. All error bars represent the standard deviations.

The crystallinity of fibers was assessed by imaging single fibers grown in a microfluidic chamber using Total Internal Reflection Fluorescence (TIRF) microscopy (**Figure** **3**a–e). For this, the different bacterial strains were inoculated in 6 µm‐high chambers with nutrients and a fluorescent cellulose‐binding dye (Fluorescent Brightener 28, Figure 3a). After 24 hours of incubation, isolated fibers were imaged and processed using Super‐Resolution Radial Fluctuations (SRRF, ImageJ plugin, Figure 3b).^[^ ^35^, ^36^ ^]^ The processed images revealed an alternating pattern of bright and dark domains, which indicate different levels of molecular packing along the fiber length. The bright domains correspond to the amorphous domains since these take up the dye more efficiently than the crystalline regions (Figure 3c). The observed pattern supports the two‐phase model of the structure of cellulose fibers.^[^ ^37^ ^]^ By extracting the intensity profiles and peaks along the fibers, we quantified the length of the amorphous (bright) and crystalline (dark) domains with a resolution of 100 nm (Figures S1 and S2, Supporting Information).

Image analysis revealed that fibers grown by the Evolved strain feature longer crystalline domains compared to other strains (Figure 3d). Indeed, the crystalline domains within Evolved fibers were found to reach a length of ≈600 nm, as opposed to 510–540 nm for other strains. Besides longer crystals, fibers produced by the Evolved strain also displayed longer amorphous domains. The amorphous domains in Evolved fibers were ≈192 nm long, whereas the other strains showed amorphous regions in the size range 167–180 nm. Due to the simultaneous increase in crystalline and amorphous domain sizes, the fraction of the crystalline phase in the Evolved fibers remained comparable to that of the other strains at 74–77% (Figure 3e). This crystallinity level falls within the range typically reported for cellulose fibers produced by bacteria.^[^ ^38^, ^39^ ^]^ The measured crystalline domain sizes are also in line with the typical range of 100–2000 nm expected for cellulose fibers.^[^ ^40^ ^]^

Beyond individual fibers, the cellulose samples produced by different bacterial strains were also systematically characterized at the level of macroscopic pellicles. Two sets of samples were prepared and examined by WAXS, electron microscopy, and mechanical testing. For one sample set, pellicles grown by distinct bacteria were washed with ultrapure water. Another set of samples was thoroughly washed in a 0.1 m NaOH solution to remove the cells, which is the standard procedure used to obtain pure bacterial cellulose. Pellicles treated with both methods were analyzed because some applications require cell removal while others might be tolerant to the presence of cells. To check whether the NaOH‐based washing procedure was effective in removing cells, we imaged the two sets of samples in a confocal microscope using pellicles that originally contained engineered cells that express Red Fluorescent Protein (RFP) to enable their visualization. The confocal images confirmed the presence of a high density of cells in the pellicles washed in water and the removal of such cells in pellicles washed with the NaOH solution (Figure S3, Supporting Information).

The crystallinity of cellulose in macroscopic pellicles washed with water or NaOH solution was measured by performing WAXS experiments across the thickness of freeze‐dried samples (Figure 3f–i). To acquire high‐quality diffractograms, samples were mounted at a 50 mm distance from the WAXS detector and measured for 30 min (Figure 3f). The 2D diffraction data did not show any orientation and could thus be radially integrated to obtain 1D diffractograms (Figure S4, Supporting Information). The obtained diffractograms reveal the peaks expected for cellulose I crystals, as well as the broad background signal associated with the presence of an amorphous phase (Figure 3g,h). Although bacterial cellulose is known to contain cellulose Iα and Iβ allomorphs,^[^ ^41^ ^]^ the Miller indices used to annotate the peaks refer to the lattice planes of cellulose Iβ (monoclinic unit cell) for simplicity. To estimate the crystallinity of the pellicles, we integrated the areas under the diffraction peaks arising from the crystals and the areas under the broad background associated with the amorphous domains between diffraction angles of 10° and 45°.

The results indicate that the crystallinity of cellulose at the pellicle level is statistically comparable among the different strains, regardless of the washing procedure used. Notably, the fraction of crystals in pellicles washed in water significantly increased from 17–20% to 25–28% after treatment with NaOH (Figure 3i). This increase in crystallinity results from a reduction in the fraction of the amorphous phase of these pellicles, as reflected by the lower background intensity at small angles measured for NaOH‐washed samples (Figure 3h; Figure S5, Supporting Information). The lower amorphous content of such pellicles can partly be explained by the removal of bacteria, which contribute to the low‐angle background pattern observed in the WAXS diffractograms (Figure S6, Supporting Information). We note that the crystalline fractions measured at the pellicle level (17–28%) are significantly lower than those estimated by image analysis at the fiber level (74–77%). This suggests the presence of an amorphous matrix and possibly remaining cells between cellulose fibers within the pellicle. In addition to crystallinity, the WAXS data were also used to estimate the lattice spacing and size of the crystalline domains (Figure S7, Supporting Information).^[^ ^42^ ^]^ The lattice spacings (2.5–6.0 Å) were found to lie in the range expected for cellulose I crystals (3–8 Å),^[^ ^43^, ^44^ ^]^ and the width of the crystalline domains (10.5–16.0 nm) falls within the typical range for bacterial cellulose (7–32 nm).^[^ ^43^, ^45^, ^46^, ^47^, ^48^, ^49^ ^]^ Such analysis revealed statistically significant small changes in crystallite size (≈1–2 nm) and lattice spacing (≈0.01–0.02 Å) after NaOH washing. The lattice spacings were also found to vary slightly (< 0.05 Å) between strains, whereas no strain dependence was observed for the width of crystalline domains.

**Figure 4:**
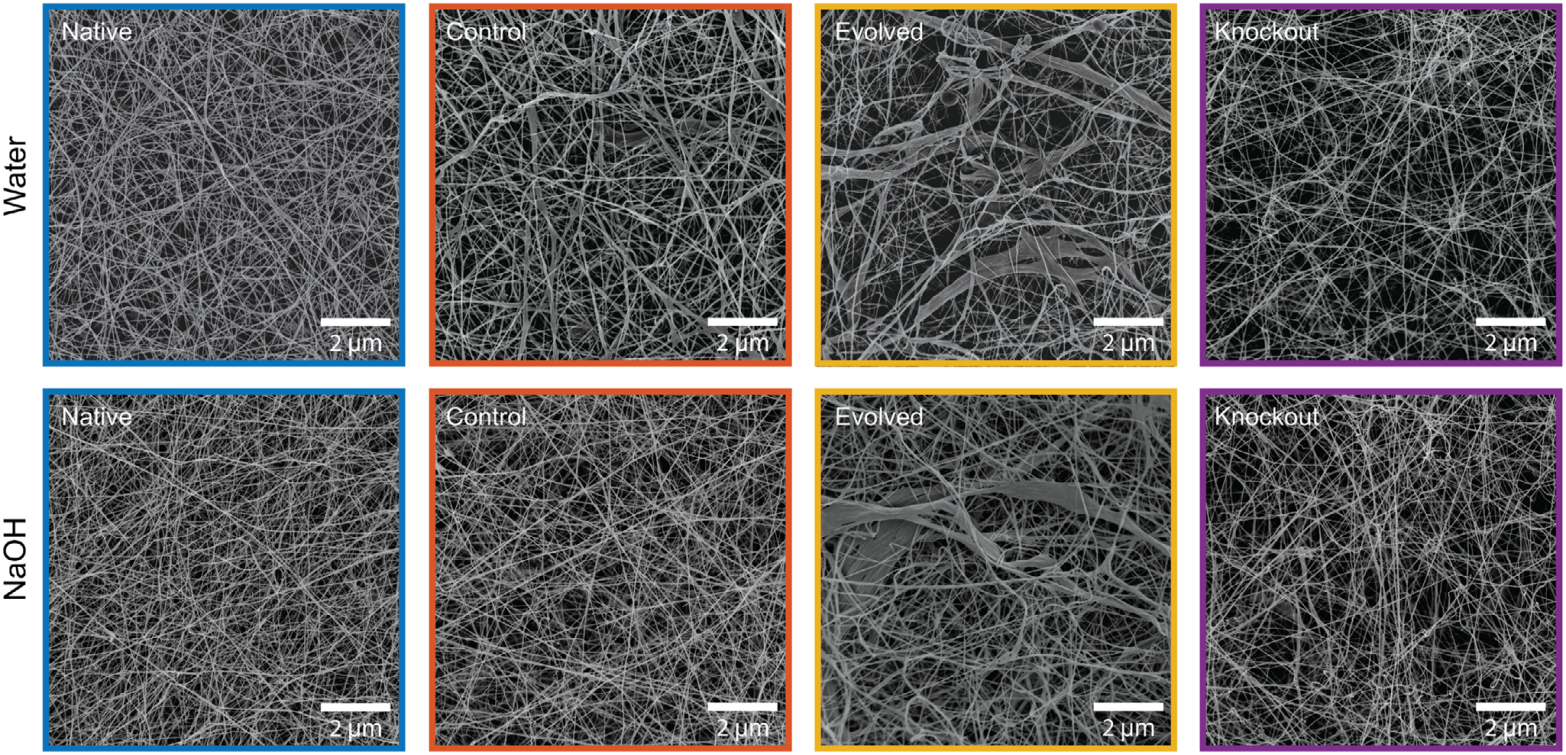
Microstructure of bacterial cellulose pellicles produced by the different *K. sucrofermentans* strains. Representative SEM images of freeze‐dried bacterial cellulose pellicles produced by the distinct bacteria. The pellicles were either washed with water (top row) or NaOH (bottom row) and then freeze‐dried. Lower magnification images are shown in Figure S10 (Supporting Information).

To gain deeper insight into the microstructure of cellulose fibers at the pellicle level, wet films produced by the distinct strains were imaged in a scanning electron microscope (SEM) after washing with water or NaOH and freeze‐drying (**Figure** **4**). Freeze‐drying was employed to preserve the network of cellulose fibers present in the liquid pellicles, thus providing information about the material's microstructure in the wet state. This drying procedure prevents the collapse of the porous network and the loss of interfibrillar space, avoiding the pellicle densification process typically observed when the samples are treated with ethanol and air‐dried (Figure S8, Supporting Information).^[^ ^51^, ^52^ ^]^

Representative SEM images of washed and freeze‐dried pellicles revealed that the Evolved strain formed a unique porous structure consisting of a hierarchical network of randomly oriented thin and thick cellulose fibers (Figure 4; Figure S9, Supporting Information). In contrast to this multiscale architecture, the Native, Control, and Knockout samples exhibit a network of fine cellulose fibers typically observed in pellicles grown at the air–water interface of the culture medium. Importantly, the thick cellulose fibers present in Evolved pellicles remain in the network even after washing the samples with NaOH solution.

Researchers have proposed that thick fibers may form in bacterial cellulose pellicles when microfibrils produced by Bcs complexes from operon *bcsI* are bound together by a cellulosic phase synthesized by Bcs complexes from operons *bcsII* and *bcsIII*.^[^ ^28^, ^53^ ^]^ According to this model, the bacterial cellulosic phase plays an analogous role to the hemicellulose, lignin, and pectin molecules that bind cellulose microfibrils in the secondary walls of plant cells. Notably, our proteomic analysis revealed that the abundance of BcsAB from operon *bcsII* produced by the Evolved bacteria is fourtimes higher than that synthesized by the Native strain (Figure 2d). The overproduction of this BcsAB protein might therefore be the reason for the thicker cellulose fibers observed in Evolved pellicles. However, the fact that BcsAB from operon *bcsII* was also present in high abundance in the Knockout strain indicates that this effect alone is not sufficient for the formation of thicker cellulose fibers (Figure 2d).

**Figure 5:**
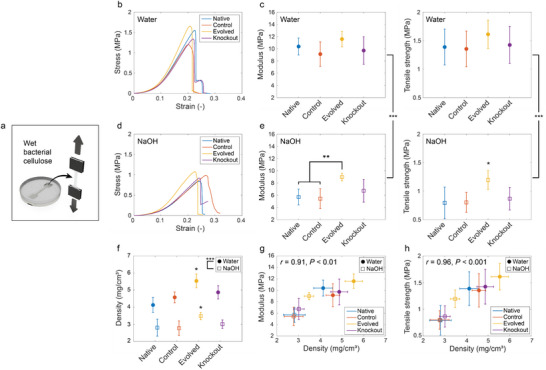
Mechanical properties of bacterial cellulose pellicles produced by the different *K. sucrofermentans* strains. a) Dogbones punched from wet bacterial cellulose pellicles were used in uniaxial tensile tests. b,d) Representative engineering stress–strain curves resulting from uniaxial tensile tests on wet cellulose pellicles from the different bacterial strains, either washed with water (b) or 0.1 m NaOH (d). c,e) Moduli and tensile strength of the different bacterial cellulose samples either washed with water (c) or NaOH (e). The moduli were calculated from the stress–strain curves between 60% and 80% of the strain at maximum stress. f) Density of the wet cellulose pellicles prepared with different bacteria and washing procedures. g,h) Effect of density on the (g) modulus (h) and tensile strength of wet pellicles produced by the distinct microorganisms. Pearson's correlation coefficients (*r*) are indicated on the graphs. Statistics: ^*^ *P* < 0.05, ^**^ *P* < 0.01, ^***^ *P* < 0.001, *n_pellicles_* ≥ 6. Error bars represent the standard deviations.

To understand the effect of the fiber network on the mechanical behavior of the pellicles, we measured the tensile properties of samples produced by the distinct bacteria (**Figure** **5**). Uniaxial tensile tests were performed on wet dogbone samples grown in culture medium for 18 days and washed with water or NaOH solution (Figure 5a). Representative stress–strain curves reveal that all pellicles displayed linear response for strains above ≈15%, followed by an abrupt rupture upon stretching beyond 20–25% strain (Figure 5b,d). To compare the mechanical properties of the distinct pellicles, we calculated the modulus of the samples from the slope within the linear stress–strain response and the tensile strength of the samples from the stress applied at rupture.

With tensile strength of 1.36–1.61 MPa and modulus in the range 9.14–11.6 MPa, the pellicles washed with water were found to be 1.35–1.75 fold stronger and 1.29–1.82 fold stiffer than pellicles washed with NaOH solution, respectively (Figure 5c,e). The *p*‐value lower than 0.001 obtained for at least six tested samples confirms the statistical relevance of the data. Notably, NaOH‐washed pellicles produced by the Evolved bacteria showed strength and stiffness comparable to those of water‐washed samples and significantly higher than those of NaOH‐washed films made with the other investigated strains. Such difference in the mechanical properties of samples produced by distinct strains was not observed among pellicles washed with water.

We hypothesize that the mechanical properties of the cellulose pellicles are controlled by the density of fibers in the sample. To test this hypothesis, we estimated the density of all pellicles by measuring the dry weight and volume of wet samples produced by the different strains (Figure 5f). The obtained results reveal that pellicles generated by the Evolved bacteria show density levels significantly higher than those produced by the other variants. This means that the overproduction of cellulose by the Evolved microorganism enables stronger densification of the pellicle rather than a simple increase in sample thickness. While such densifying effect occurs in both sets of washed samples, the results suggest that the removal of cells and potentially amorphous cellulosic phase during NaOH‐washing strongly reduces the pellicle density from 4.13–5.54 to 2.77–3.48 mg cm^−3^. The swelling of pellicles upon NaOH washing additionally contributes to this decrease in density (Figure S11, Supporting Information).

By comparing the above data with the strength and modulus measurements, we found a strong correlation between the mechanical properties and the density of the pellicles (Figure 5g,h). This validates the initial hypothesis and highlights the importance of fiber density in forming pellicles with a strong and stiff percolating network. By forming a dense hierarchical network of fibers, the Evolved bacteria are able to produce pellicles that are both stronger and stiffer than those made by the other microorganisms. While further research is needed to elucidate structure‐property relationships of such networks, the presence of thicker ribbons within a finer fiber network may potentially be tailored to further enhance the mechanical properties of the pellicle by enabling a more homogeneous stress distribution through the material upon mechanical loading (Figure 4).^[^ ^54^ ^]M^

The higher fiber concentration and the hierarchical architecture of the Evolved pellicles compensate for the loss in the cell fraction and potentially amorphous phase resulting from the washing procedure with NaOH. Since washing with NaOH is a common procedure to remove bacteria in biomedical and textile applications, the high strength and stiffness achieved with the Evolved bacteria represent a major advantage of this strain for the biofabrication of cellulose pellicles.

## 3 Conclusion

*K. sucrofermentans* evolved to overproduce cellulose can generate cell‐free pellicles with a hierarchical fiber network that is denser and stronger compared to those made by native microorganisms. The unique structure and mechanical properties of the Evolved pellicles emerge from a 12‐base pair deletion in a gene encoding the protease subunit ClpA. This small and simple genetic mutation has a major impact across the entire bacterial proteome, which in turn determines the structure and mechanics of the final cellulose pellicles. Proteomic analysis shows that the 12‐bp deletion changes the abundance of a broad range of proteins that directly or indirectly affect the biological machinery used by the bacteria for the extrusion and assembly of cellulose fibers. While some of the changes in protein abundance are expected to favor cellulose formation, other alterations in the proteome reveal effects that could not be anticipated considering single gene‐trait links alone. On the one hand, the strong up‐regulated expression of a bacterial cellulose synthase sub‐unit (BcsAB_2) and of a porin (OprB) clearly contributes to cellulose formation by enhancing the abundance of proteins associated with the fiber extrusion machinery and by increasing the intracellular availability of sugar molecules, respectively. On the other hand, the up‐regulated expression of some of the proteins associated with the competitive synthesis of gluconic acid (Gcd) and with the activation of the cellulose synthase machinery (DosP) represent unexpected effects that can only be rationalized if a broader range of genes and proteins are considered in the analysis. Combined, these changes in the proteome landscape favor cellulose overproduction and the formation of thick cellulose fibers within the network of thin fibrils in samples produced by the Evolved strain. The lower cellulose production and the absence of thick fibers in Knockout pellicles indicate that a lack of ClpA‐ClpS interactions is not sufficient to explain the overproducing phenotype of the Evolved fiber network. Further metabolomic studies could help elucidate how the metabolome of genetically distinct strains is connected to cellulose overproduction. After removal of the cells, the dense hierarchical fiber network generated by the Evolved bacteria is 57% stiffer and 51% stronger than pellicles produced by the Native strain. The differential protein expression patterns arising from a seemingly unrelated genetic mutation highlight the value of the directed evolution approach as a compelling experimental tool that dispenses prior knowledge on gene‐trait links to identify microorganisms for biofabrication and other microbial biotechnologies.

## 4 Experimental Section

### Bacterial Strains

*Komagataeibacter sucrofermentans* JCM 9730 (American Type Culture Collection, ATCC 700178, SAMN37928908) is the Native cellulose‐producing bacterial strain used in this study. The Control, Evolved, and Knockout strains were obtained from previous research.^[^ ^9^ ^]^ Briefly, the Control strain is genetically identical to the Native strain but has been stressed by external factors, such as lack of nutrients and light. The Evolved strain JML 2321 (SAMN37928909) comes from a directed evolution process of the Native strain, and it has a 12‐bp deletion in the genes coding for a protease (ClpAPS), more precisely in the *clpA* gene at the binding site with *clpS*. The Knockout strain JML KO 23 (SAMN37928910) was obtained by knocking out *clpS* from the Native strain. More precisely, the start codon of *clpS* was deleted to minimally disrupt the downstream expression of *clpA*. Indeed, in *E. coli*, a *clpA* promoter appears in the *clpS* coding region.^[^ ^55^ ^]^ As the organization of those genes is similar in *K. sucrofermentans*, it was reasoned that something similar might exist in the strain because *clp* sequences are known to contain highly conserved elements.^[^ ^24^ ^]^ By deleting only the start codon, the disruption of the clpA sequence could be minimized whilst ensuring a knockout. All strains grow in a medium composed of 25 g L^−1^ D‐mannitol (Thermo Fisher Scientific), 5 g L^−1^ yeast extract (Sigma–Aldrich), 3 g L^−1^ peptone (Sigma–Aldrich), and optionally 15 g L^−1^ agar (Sigma–Aldrich) for solid medium.

### RFP‐Expressing Strain Generation

100 µL of electrocompetent Native *K. sucrofermentans* cells were transformed with plasmid J23104‐mRFP1‐331Bb (Addgene plasmid #78274)^[^ ^1^ ^]^ by electroporation using a MicroPulser (Bio‐Rad) with an 0.2 cm cuvette, using the Ec2 (2.5 kV) setting. Cells were then allowed to recover overnight in 1 mL medium containing 2 vol% cellulase at 28 °C shaking at 225 rpm. The following morning, 250 µL of the resultant culture was plated onto 1.5% agar plates containing medium and 185 µg mL^−1^ chloramphenicol and allowed to grow for several days. The resultant colonies appeared red and had strong RFP fluorescence.

### Bacterial Cellulose (BC) Production

Frozen stocks of *K. sucrofermentans* (−80 °C) were thawed, streaked onto solid medium and incubated for 7 days to isolate single colonies (28 °C, static, 80% relative humidity (RH)). Bacterial cellulose pellicles were grown from those single colonies. Colonies were inoculated into 5 mL of liquid medium in 50 mL Falcon tubes (Techno Plastic Products AG, TPP) closed with a foam plug, and incubated for 12 days (28 °C, static, 80% RH) to form BC pellicles at the air–medium interface (if not stated otherwise). Earlier research has shown that the pellicles formed by the Native strain grow mostly within the first 4 days of incubation, beyond which their weight remains approximately constant.^[^ ^9^ ^]^ After the incubation time, the cellulose pellicles were retrieved and washed three times in 50 mL ultra‐pure water (MilliQ). Some of the pellicles were then immersed in 50 mL 0.1 m NaOH for 1 hour at room temperature (RT) to remove cells and then brought back to neutral pH using sequential washes of ultra‐pure water. The samples were either annotated “water” if only washed with ultra‐pure water or “NaOH” if they were treated with NaOH. The NaOH‐washed pellicles were weighed using a precision balance (UMT2 Microbalance, Mettler Toledo) to measure the cellulose quantity produced by each strain.

### Proteomics

*Protein Extraction*: Bacteria were grown to their exponential phase (3 days of incubation), flash‐frozen in liquid nitrogen, and kept at −80 °C until further use. To concentrate the cells and cellulose, the cultures were thawed, vortexed, spun down at 4500 rcf for 10 min, and the supernatant was removed. The pellet was resuspended in 5 mL of a lysis buffer composed of 10% sodium dodecyl sulfate (SDS, Sigma–Aldrich) and 100 mm 4‐(2‐hydroxyethyl)‐1‐piperazineethanesulfonic acid (HEPES, Sigma–Aldrich) in water. To lyse cells and remove large cellulose pieces, the samples were tip sonicated for 3 min at maximum power (Bandelin Sonopuls), bead beaten (five times 30 s, 10 s pause, MP FASTPREP‐24), and syringe filtered (0.2 µm mesh). After this process, the lysates were centrifuged at 21 300 rcf for 10 min to remove insoluble debris. The protein concentration was quantified in all samples with a BCA assay (bicinchoninic acid assay, Thermo Fisher Scientific). Lysates were adjusted to 0.4 mg mL^−1^ protein in the lysis buffer. Triplicates were prepared for the following steps.

*Protein Reduction, Alkylation, and Digestion*: Proteins were reduced with 5 mm TCEP at 55 °C for 15 min, alkylated with 20 mm MMTS at room temperature for 10 min and then acidified to pH < 1 with phosphoric acid before trapping them in S‐Trap micro spin columns (ProtiFi, LLC). The S‐Trap micro spin column manufacturer protocol was used to clean the proteins, digest them with Trypsin, and elute the peptides. The peptides were lyophilized and stored at −80 °C until further use.

*Mass Spectrometry Data Acquisition*: Lyophilized peptides were dissolved at 1 µg µL^−1^ in 0.1% formic acid (Sigma–Aldrich) and analyzed by nLC‐MS/MS for MS analysis. Peptides were loaded onto a 100 µm ID × 3 cm precolumn packed with Reprosil C18 3 µm beads (Dr. Maisch GmbH), and separated by reverse‐phase chromatography on a 100 µm ID × 35 cm analytical column packed with Reprosil C18 1.9 µm beads (Dr. Maisch GmbH) and housed into a column heater set at 50 °C. DIA‐MS measurements were performed on an Orbitrap Exploris 240 Mass Spectrometer (Thermo Fisher) equipped with a Vanquish Neo nanoLC system (Thermo Fisher). Peptides were separated by a 60‐min effective gradient ranging from 6 to 30% acetonitrile in 0.125% formic acid. All samples were measured in DIA mode. The DIA‐MS method consisted of a survey MS1 scan from 350 to 2000 m/z at a resolution of 120000 with an AGC target of 50% or 100‐ms injection time, followed by DIA in 41 variable‐width isolation windows. Precursors were isolated by a quadrupole and fragmented with HCD with a collision energy of 28%. MS2 spectra were acquired with a scan range of 200 to 1800 m/z at an orbitrap resolution of 30 000 with an AGC target of 200% or 54‐ms injection time.

*Mass Spectrometry Data Analysis*: Annotated genomes of bacterial strains (NCBI: SAMN37928908) were used to generate a reference protein fasta database. DIA‐NN within the Fragpipe environment^[^ ^56^ ^]^ was used to search DIA data using standard settings. R^[^ ^57^ ^]^ with the “tidyverse” package collection^[^ ^58^ ^]^ was used for all analyses. Differential protein expression was determined using the LIMMA package^[^ ^59^ ^]^ on all samples at once. Significant differential expression between all strains was then calculated, and *P*‐values were corrected globally using Benjamini‐Hochberg correction. Heatmaps were generated using MATLAB R2023a (MathWorks).

### Raman Spectroscopy

The chemical composition of the freeze‐dried bacterial cellulose (BC) pellicles was analyzed using a confocal Raman spectroscope (LabRam Soleil, Horiba Scientific, France). A 532 nm (λ) linearly polarized excitation laser with a nominal power of 77 mW at the sample was used for the measurements. The excitation laser was focused on the sample using a 100×, 0.9 NA objective lens (Nikon, Japan). This setup allowed clear visualization of the sample with a diffraction‐limited laser spot size of ≈721 nm ($\frac{1.22\;\lambda }{NA}$). A confocal pinhole with a 50 µm diameter was employed to exclude any bacteria from the probe volume. Raman measurements were conducted using a grating with 600 lines/mm with an acquisition time of 5 min per spectrum. Three measurements per sample were recorded, carefully avoiding areas showing bacterial presence. Raman spectra were processed using LabSpec 6.7.1.10 software (Horiba Scientific, France), which involved smoothening of the spectra using Savitzky–Golay filter (polynomial degree: 7, window size: 12 points), polynomial background subtraction (polynomial degree: 8, window size: 256 points) and normalization to the most intense Raman peak at 1094 cm^−1^.

### Single‐Fiber Analysis

*Microfluidic Device*: Microfluidic chambers of 6 µm height were prepared using standard photolithography and soft lithography methods. Briefly, a SU‐8 photoresist (3000 series, MicroChem) was patterned on a silicon wafer in the cleanroom and used as a master for soft lithography (1:10 polydimethylsiloxane (PDMS), Sylgard 184, Dow Corning). After curing, the chambers were peeled off, an inlet hole was punched, and the device was bonded to a glass slide using air plasma (20 s, 0.4 mbar, medium level, Plasma Cleaner PDC‐32G). The microfluidic chambers were sterilized using UV‐C (180 mJ cm^−2^, 254 nm, UVP Cross‐linker CL‐3000, AnalytikJena) and then filled with cellulose‐producing bacteria culture diluted from frozen stocks (≈1.8×10^6^ CFU mL^−1^). To visualize the cellulose fibers, the medium also contained a cellulose‐binding fluorescent dye (218 µm Fluorescent Brightener 28, Sigma–Aldrich). The filled chambers were incubated 24 hours before imaging (28 °C, static, 80% RH).

Microscopy: Total Internal Reflection Fluorescence (TIRF) microscopy (Leica DMI6000B inverted) enabled the imaging of single bacterial cellulose fibers. A 405 nm laser was used in combination with a 100x objective (1.47 NA Oil HCX PlanApo) to image single fibers, which were visible thanks to the fluorescent dye. Timelapses were taken (100 frames, 300 ms exposure, 512×512 resolution) with the following filters: external excitation filter of 405/20, Quad cube 405/10‐418‐450/55 (excitation‐dichroid‐emission), external emission filter of 450/45.

*Image Analysis*: All images were processed with the Super‐Resolution Radial Fluctuations (SRRF) NanoJ plugin^[^ ^35^ ^]^ from ImageJ^[^ ^36^ ^]^ (Figure S1a,b, Supporting Information). The following parameters were used: temporal radiality auto‐correlations (TRAC), 0.5 ring radius, 10 radiality magnification, and 8 axis in ring, with a drift‐correction. On the processed images, 3 µm wide spline lines were fitted along the traced isolated fibers using the segmented line tool (Figure S1c, Supporting Information), and an intensity profile was generated along every fiber. Using *findpeaks* (MATLAB R2023a, MathWorks), the peaks were identified, and their full‐width half maximum (FWHM) and height were measured (Figure S1d, Supporting Information). A minimum peak FWHM and peak spacing of 100 nm were set, as this is the resolution of TIRF microscopy (Figure S1e, Supporting Information). Additionally, the median peak prominence was calculated for each fiber and used as a threshold to discard all peaks with a lower prominence than the median (Figure S1f, Supporting Information). The length of the bright (more amorphous) regions corresponded to the FWHM of the peaks and the length of the dark regions (more crystalline) corresponded to the distance between adjacent peaks at the half maximum intensity. Crystallinity was then calculated using the following equation for each image:

(1) $$crystallinity\left(\%\right)=\frac{\sum dark\;lengths}{\sum dark\;lengths+\sum bright\;lengths}\times 100$$

### Wide‐Angle X‐Ray Scattering (WAXS)

For WAXS measurements, 4 mm diameter samples were punched out from freeze‐dried BC pellicles (disposable biopsy punch, Kai Europe GmbH), and mounted onto a solid sample holder (Xenocs). The measurements were performed with a laboratory SAXS/WAXS system (Xenocs Xeuss 3.0), using Cu Kα radiation (1.54 Å, Xenocs Genix 3D micro source) with a beam diameter of 1 mm and a sample‐detector distance of 50 mm (Dectris EIGER2 Si 1M, pixel size 75 µm × 75 µm), calibrated using a Lanthanum hexaboride standard. Each sample was measured under vacuum for 30 min. More precisely, the “line eraser” setting was activated, which means two diffractograms of 15 min were recorded with a slightly shifted detector position, and subsequently merged to remove the slit of the detector, which would otherwise interfere with the integration. All data handling was performed using the Xenocs XSACT software (version 2.9, Xenocs). The 1D scattering data was calculated by azimuthally integrating the 2D signal. The “visual peak search” function was used to fit an amorphous background to the 1D diffractograms and to fit peaks to extract their position and full width at half maximum (FWHM). The crystallinity index was calculated between 2θ=10° and 2θ=45°, using the following equation:

(2) $$crystallinity\left(\%\right)=\frac{area\;sample-area\;background}{area\;sample}\times 100$$

The lattice spacing (d) was estimated using Bragg's equation $d=\frac{2n\pi }{q}$ where n=1 is the diffraction order, and q is the scattering vector. The crystallite size (τ) was estimated using Scherrer's equation $\tau =\frac{K\lambda }{\beta \cos\theta }$ where K=0.94 is the shape factor, β is the FWHM, and θ is the Bragg angle.

### Scanning Electron Microscopy (SEM)

To compare the microstructure of the BC pellicles, 4 mm diameter samples were punched out from freeze‐dried samples (disposable biopsy punch, Kai Europe GmbH) and deposited on SEM stubs, with the top of the pellicle facing up. Another set of samples was prepared from pellicles washed with increasing ethanol solution concentrations (10–100%; 10% steps) before air‐drying. The stubs were coated with 4 nm of platinum (compact coating unit CCU‐010, Safematic), and silver paste was added to the side of the sample to limit charging (G 3692 silver DAG, Plano GmbH). Images were taken with an InLens detector, an accelerating voltage of 3 kV, and an aperture of 500 pA (GeminiSEM 450, Carl Zeiss Microscopy GmbH). The images were line‐averaged (*n* = 8), and their resolution was 3072×2304 pixels.

### Uniaxial Tensile Tests

To assess the mechanical properties of the cellulose produced by the different strains, uniaxial tensile tests were performed on bigger BC pellicles. To prepare those pellicles, 100 mL of medium in 500 mL Erlenmeyers were inoculated with each strain (4×10^5^ CFU/mL) and grown for 18 days (28 °C, static, 80% RH). The pellicles were washed, as explained earlier, with ultra‐pure water or sodium hydroxide (NaOH). Dogbones (2 mm width) were punched out from the wet pellicles, put back into ultra‐pure water, and taken out right before tensile testing on the same day. The dogbones were clamped into a texture analyzer (TA.XTplus Texture Analyzer, Stable MicroSystems) with a starting spacing of 20 mm. The thickness of the samples was measured with a caliper once mounted. A displacement rate of 1.2 mm min^−1^ and a 500 g load cell were used, and the test was stopped after the dogbones broke. A minimum onset of 2.5 mN was used to shift all curves. The reported moduli were calculated from the linear region between 60% and 80% of the strain at maximum stress for each sample.

### Density Determination

To determine the density of BC pellicles, both their volume and dry weight were measured. After removing excess water, pictures of the side view of pellicles in a wet state were taken with a camera (Canon EOS 6D; Canon Macro 100 mm objective). A 1 mm‐thick glass slide was incorporated into the image to scale the picture. The cross‐sectional areas of the pellicles were estimated with ImageJ^[^ ^36^ ^]^ thresholding. By equating those calculated areas $A_{\mathrm{measured}}$ to the area of a cylinder cross‐section $A_{\mathrm{cylinder}}=2\mathrm{rh}$, h could be calculated as $h=\frac{A_{\mathrm{mesured}}}{2r}$, r being the radius of the container in which pellicles were grown and h being the equivalent height of pellicles if they were perfect cylinders. Next, the volume of each pellicle was estimated as $V=\pi r^{2}h$. The same pellicles were then air‐dried and weighed with a precision balance (UMT2 Microbalance, Mettler Toledo) to obtain the pellicle mass W. Density was finally calculated as $\rho =\frac{W}{V}$.

### Statistical Analysis

All statistical analysis was performed using MATLAB R2023a (MathWorks). To compare multiple groups (Native, Control, Evolved, and Knockout strains), one‐way ANOVAs were conducted. Alternatively, two‐way ANOVAs were conducted to compare strains (Native, Control, Evolved, Knockout) and washing methods (Water, NaOH). If the result showedk a statistically significant difference between the groups (*P* < 0.05), a pairwise comparison was made with a Bonferroni correction to compensate for the effects of multiple comparisons. Statistical analysis of the proteomic data was performed as explained in the Proteomics subsection above.

## Conflict of Interest

The authors declare the following competing interests: two patent applications were submitted based on some of the results reported in this research.

## Author Contributions

A.R.S. and J.M.L. conceptualized the research project; J.M.L., M.S., A.K., M.R., M.L., S.B., D.H., N.K., and A.R.S. designed the experiments; J.M.L., M.R., M.L., S.B., and D.H. conducted the experiments; A.K. engineered the Knockout strain; J.M.L. produced all the bacterial cellulose samples; M.L. prepared, measured, and analyzed the samples for the proteome analysis with the support of J.M.L. and M.S.; S.B. performed the Raman spectroscopy experiments and analyzed the data; J.M.L. produced the microfluidic devices, prepared the cellulose single‐fiber samples, and analyzed the images; M.R. performed the X‐ray experiments and analyzed them with the support of J.M.L.; J.M.L. took the scanning microscopy images with the support of M.S.; D.H. measured and analyzed the mechanical properties with the support of J.M.L.; J.M.L. measured and analyzed the density; J.M.L. and A.R.S. wrote the manuscript draft and prepared the figures; J.M.L., M.S., A.K., M.R., M.L., S.B., D.H., N.K., and A.R.S. discussed the results and contributed to the final version of the manuscript; J.M.L., M.R., M.L., S.B., and A.R.S. wrote the paper.

## Supporting information



Supporting Information

## Data Availability

The data that support the findings of this study are available from the corresponding author upon reasonable request.
