# Supplementary material for: Genetic Impacts on the Structure and Mechanics of Cellulose Made by Bacteria
Source: Adv Sci (Weinh). 2025 Jun 5;12(33):e05075. doi: 10.1002/advs.202505075 (PMC12412545; doi:10.1002/advs.202505075)
Supplement: Supplementary file 1 — Supporting Information [file ADVS-12-e05075-s001.docx]

**Supporting Information**

**Genetic impacts on the structure and mechanics of cellulose made by bacteria**

Julie M. Laurent,^1^ Mathias Steinacher,^1^ Anton Kan,^1^ Maximilian Ritter,^2,3^ Mario Leutert,^4^ Siiri Bienz,^5^ David Häberlin,^1^ Naresh Kumar,^5^ André R. Studart^1^*

^1^ Complex Materials, Department of Materials, ETH Zürich, 8093 Zürich, Switzerland

^2^ Wood Materials Science, Institute for Building Materials, Department of Civil, Environmental and Geomatic Engineering, ETH Zürich, 8093 Zürich, Switzerland

^3^ WoodTec group, Cellulose and Wood Materials, Empa – Swiss Federal Laboratories for Materials Science and Technology, 8600 Dübendorf, Switzerland

^4^ Institute of Molecular Systems Biology, Department of Biology, ETH Zürich, 8093 Zürich, Switzerland

^5^ Laboratory of Organic Chemistry, Department of Chemistry and Applied Biosciences, ETH Zürich, 8093 Zürich, Switzerland

* corresponding author

**Content:**

Supporting Tables S1, S2

Supporting Figures S1-S11

References

**Supporting Tables**

**Table S1. Selection of differential protein expressions in the Evolved and Knockout strains that may indirectly affect the production of cellulose by *K. sucrofermentans*.** Only proteins showing a statistically significant differential protein expression compared to the Native strain are shown. In the regulation columns, empty rows (“/”) indicate that no statistical differences in differential protein expression were found. Statistics: **P* < 0.05, ***P* < 0.01, ****P* < 0.001, *****P* < 0.0001).

| Protein | Regulation /  log_2_(fold change)  (Evolved *vs.* Native) | Regulation /  log_2_(fold change)  (Knockout *vs.* Native) | Function | Expected impact on cellulose production |
| --- | --- | --- | --- | --- |
| PQQ |  |  |  |  |
| Gcd_1 (P15877) | ***Up / 1.45 | *Up / 0.79 | PQQ-dependent glucose dehydrogenase | - [1] |
| PqqCD (Q49150) | *Down / -0.71 | *Down / -0.76 | PQQ biosynthesis | + [1] |
| c-di-GMP availability |  |  |  |  |
| DosP_6 (P76129) | **Down / -1.01 | / | c-di-GMP phosphodiesterase | + [2] |
| DosP_7 (P76129) | *Up / 0.92 | / | c-di-GMP phosphodiesterase | - [2] |
| DosP_10 (P76129) | *Up / 0.74 | *Up / 0.72 | c-di-GMP phosphodiesterase | - [2] |
| DosP_11 (P76129) | *Up / 0.66 | *Up / 0.80 | c-di-GMP phosphodiesterase | - [2] |
| Carbon source availability |  |  |  |  |
| OprB (Q51485) | **Up / 1.39 | *Up / 0.99 | Sugar transmembrane transport | + [3] |
| GalP (P0AEP1) | **Up / 0.79 | *Up / 0.75 | Galactose-proton symporter | + [4] |
| Cell resistance |  |  |  |  |
| AlsD (Q04777) | ***Up / 1.62 | *Up / 0.76 | Alpha-acetolactate  (controls internal pH of cells during stationary phase) | + [5] |
| CdsA (Q9X1B7) | **Up / 1.09 | ***Up / 2.27 | Lipid/Phospholipid biosynthesis |  |
| ClsA (P71040) | *Up / 0.98 | *Up / 1.09 | Lipid/Phospholipid biosynthesis |  |
| MrdA (P0AD65) | **Up / 1.48 | **Up / 1.43 | Peptidoglycan D,D-transpeptidase  (crosslinking of cell wall, determines rod shape) |  |
| YdeP (P77561) | ***Up / 1.60 | **Up / 0.91 | Probably involved in acid resistance | + [6] |
| Gluconeogenesis/glycolysis/  pentose phosphate pathway |  |  |  |  |
| Afr_2 (Q2I8V6) | *Down / -1.02 | / | Converts fructose to mannitol |  |
| Fba (Q0PAS0) | **Up / 0.72 | / | Fructose-bisphosphate aldolase |  |
| Fda (Q07159) | *Down / -0.52 | / | Fructose-bisphosphate aldolase |  |
| MaeA (P26616) | *Up / 1.02 | / | Oxaloacetate decarboxylase | + [7] |
| NA (Q5FQ97) | *Up / 1.32 | *Down / -0.65 | Gluconokinase  (gluconate -> 6-phosphogluconate) |  |
| Ndk (Q9PIG7) | *Down / -0.96 | / | CTP/GTP/UTP biosynthetic process | - [8] |
| Pdc (P06672) | **Up / 0.72 | / | Pyruvate decarboxylase  (pyruvate -> acetaldehyde) | - [9] |
| Pgk (P18912) | *Down / -0.95 | / | Phosphoglycerate kinase  (1,3-BiP-glycerate <-> 3P-glycerate) |  |
| Pgl (P46016) | *Down / -0.70 | / | 6-phosphogluconolactonase  (6P-gluconolactone -> 6P-gluconate) |  |
| PpdK (P22983) | ***Up / 3.84 | *Up / 0.90 | Pyruvate phosphate di-kinase  (pyruvate -> phosphoenolpyruvate (PEP)) | + [7] |
| Tkt (A0A0I9QGZ2) | *Down / -0.91 | / | Transketolase  (pentose phosphate pathway) |  |
| TreF (P62601) | **Down / -1.02 | / | Cytoplasmic trehalase  (trehalose -> glucose) |  |
| YieH (P31467) | *Down / -0.73 | / | 6-phosphogluconate phosphatase  (6P-gluconate or DAHP or PEP dephosphorylation) |  |
| Zwf_2 (A0QP90) | **Up / 0.63 | / | Glucose-6-phosphate 1-dehydrogenase  (glucose-6P -> 6P-gluconolactone) |  |
| Zwf_3 (P0AC53) | *Up / 0.40 | / | Glucose-6-phosphate 1-dehydrogenase  (glucose-6P -> 6P-gluconolactone) |  |
| DNA replication |  |  |  |  |
| DnaA (P03004) | **Up / 0.90 | *Up / 0.57 | Chromosomal replication initiator |  |
| DnaE1 (B8GWS6) | **Up / 1.15 | **Up / 1.34 | DNA polymerase III subunit α |  |
| Transcription/translation |  |  |  |  |
| AadR (Q01980) | **Up / 1.35 | *Up / 1.34 | Transcriptional activatory protein (anaerobic gene expression) |  |
| DksA (P0ABS1) | *Down / -1.34 | / | Transcription factor  (down-regulates rRNA expression and up-regulates amino acid biosynthesis) |  |
| RelE (P0C077) | **Down / -1.13 | *Down / -1.01 | mRNA interferase toxin  (negative transcription regulation) |  |
| RhlE_2 (P25888) | **Up / 0.99 | *Up / 0.67 | RNA helicase  (ribosome assembly) |  |
| RpsU (Q6N274) | **Up / 1.07 | *Up / 0.87 | Small ribosomal subunit protein bS21 |  |
| MnmG (P0A6U3) | *Up / 1.05 | / | tRNA uridine 5-carboxymethylaminomethyl modification enzyme (regulation of cytoplasmic translational fidelity) |  |
| Other polymers |  |  |  |  |
| CptA (Q7CPC0) | *Up / 1.16 | / | Phosphoethanolamine transferase to the outer membrane glucan |  |
| EpsJ (P71059) | **Up / 0.90 | *Up / 0.86 | May be involved in the production of the EPS component during biofilm formation |  |
| OpgE (P75785) | **Up / 0.90 | / | Phosphoethanolamine transferase OpgE to periplasmic glucan |  |
| Stress response |  |  |  |  |
| DnaJ_3 (P50018) | ***Up / 1.25 | *Up / 0.63 | Chaperone protein in response to hyperosmotic and heat shock  (prevents protein aggregation, protein refolding) |  |
| Hmp (P39662) | **Up / 1.01 | / | Flavohemoprotein  (NO detoxification) |  |
| IbpA_3 (P0C054) | **Up / 1.26 | / | Heat shock protein  (prevents protein irreversible denaturation and proteolysis) |  |
| Lon2 (P36774) | **Up / 1.38 | *Up / 0.68 | Lon protease 2  (degrades selectively mutant or abnormal proteins after DNA damage) |  |
| NemA_2 (P77258) | ***Up / 1.22 | *Up / 0.44 | N-ethylmaleimide reductase (degradation of toxic compounds) |  |
| NrdJ (O69981) | *Up / 1.00 | **Up / 1.22 | Vitamin B12-dependent ribonucleotide reductase  (provides a pool of precursors for DNA repair during oxygen limitation and/or for immediate growth after O_2_ restoration) |  |
| YbiO_1 (P75783) | **Up / 0.82 | *Up / 0.65 | Mechanosensitive channel  (protects cells against osmotic shock) |  |
| YvyD (P28368) | **Up / 0.99 | / | Ribosome hibernation promotion factor (response to stress) |  |
| AhpC (P0A251) | **Down / -1.13 | / | Protection against oxidative stress by detoxifying peroxides |  |
| Bcp (Q83CY8) | **Down / -1.32 | *Down / -0.95 | Protection against oxidative stress by detoxifying peroxides |  |
| CspA (Q9Z3S6) | *Down / -1.86 | *Down / -1.12 | Cold shock protein  (activates transcription) |  |
| DksA (P0ABS1) | *Down / -1.34 | / | RNA polymerase-binding transcription factor  (involved in repair of double-strand DNA breaks) |  |
| Dps1 (Q9RS64) | **Down / -0.78 | **Down / -1.07 | DNA protection during starvation |  |
| MutY (P17802) | **Down / -1.64 | / | Adenine DNA glycosylase  (DNA mismatch and base-excision repair) |  |
| NemA_1 (P77258) | *Down / -0.53 | *Down / -0.52 | N-ethylmaleimide reductase (degradation of toxic compounds) |  |
| NemA_3 (P77258) | *Down / -0.56 | *Down / -0.62 | N-ethylmaleimide reductase (degradation of toxic compounds) |  |
| Pcm (A5F9C1) | **Down / -1.10 | / | Protein-L-isoaspartate O-methyltransferase  (repair and/or degradation of damaged proteins) |  |
| RelE (P0C077) | **Down / -1.13 | *Down / -1.01 | mRNA interferase toxin (mediator of cell death in liquid media, response to amino acid starvation) |  |
| SodC (P20379) | *Down / -1.18 | *Down / -1.04 | Superoxide dismutase  (destroys toxic radicals) |  |

**Table S2.** **Tentative assignment of Raman signals from cellulose and C=O.**

| Raman shift (cm^-1^) | Vibrational assignment | References |
| --- | --- | --- |
| 250-500 | Ring breathing/deformation | Makarem *et al.* [10] |
| 998-1058 | C-O or C-C stretching | Makarem *et al.* [10] |
| 1094 | Ring breathing | Lee *et al.* [11] |
| 1122-1149 | C-O-C skeletal | Lee *et al.* [11] |
| 1337-1406 | HCH, HOC, or HCC bending | Wiley *et al.* [12] |
| 1740 | C=O stretching | Zhang *et al.* [13] |
| 2329 | N_2_ gas (environmental) | Le *et al.* [14] |
| 2890 | CH stretching | Lee *et al.* [11] |
| 2940 | CH_2_ asymmetric stretching | Lee *et al.* [11] |
| 2968 | CH_2_ asymmetric stretching | Lee *et al.* [11] |
| 3248 | OH stretching | Lee *et al.* [11] |
| 3375 | OH stretching | Lee *et al.* [15] |

**Supporting Figures**


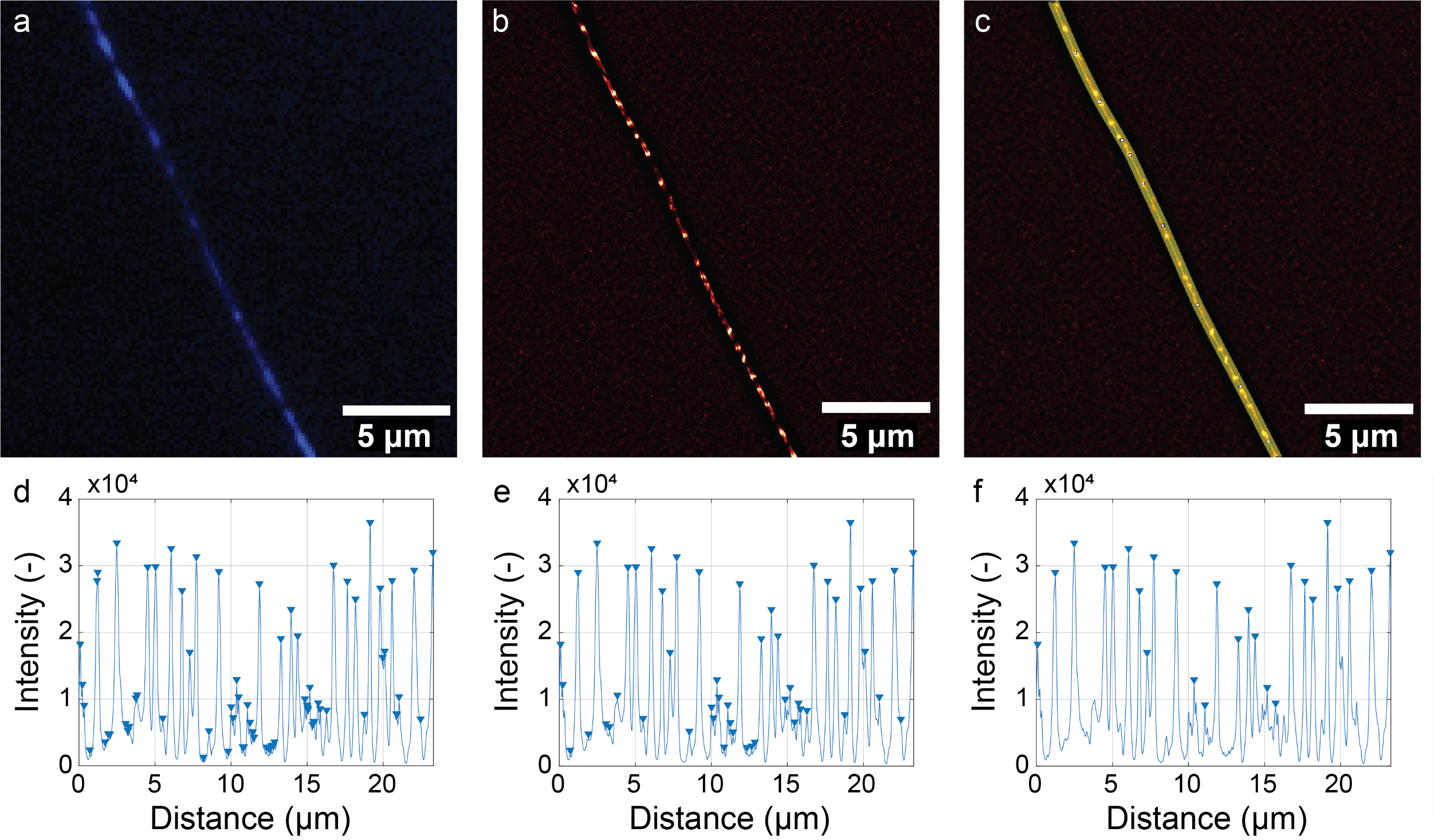


**Figure S1. Image processing of single bacterial cellulose fibers in a microfluidic chamber.** **(a)** Total Internal Reflection Fluorescence (TIRF) microscopy of a single bacterial cellulose fiber produced by the Evolved strain. **(b)** Processed image using Super Resolution Radial Fluctuations (SRRF) in ImageJ. [16] **(c)** Spline line fitted along the fiber. **(d-f)** Extracted line intensity profiles along the fiber. Peaks are indicated with blue inversed triangles and were found using *findpeaks* function in MATLAB, with (d) no constraints, (e) using a minimum peak width and peak spacing of 100 nm (TIRF resolution), or (f) using a minimum peak width and peak spacing of 100 nm (TIRF resolution) and a threshold corresponding to the median peak prominence.


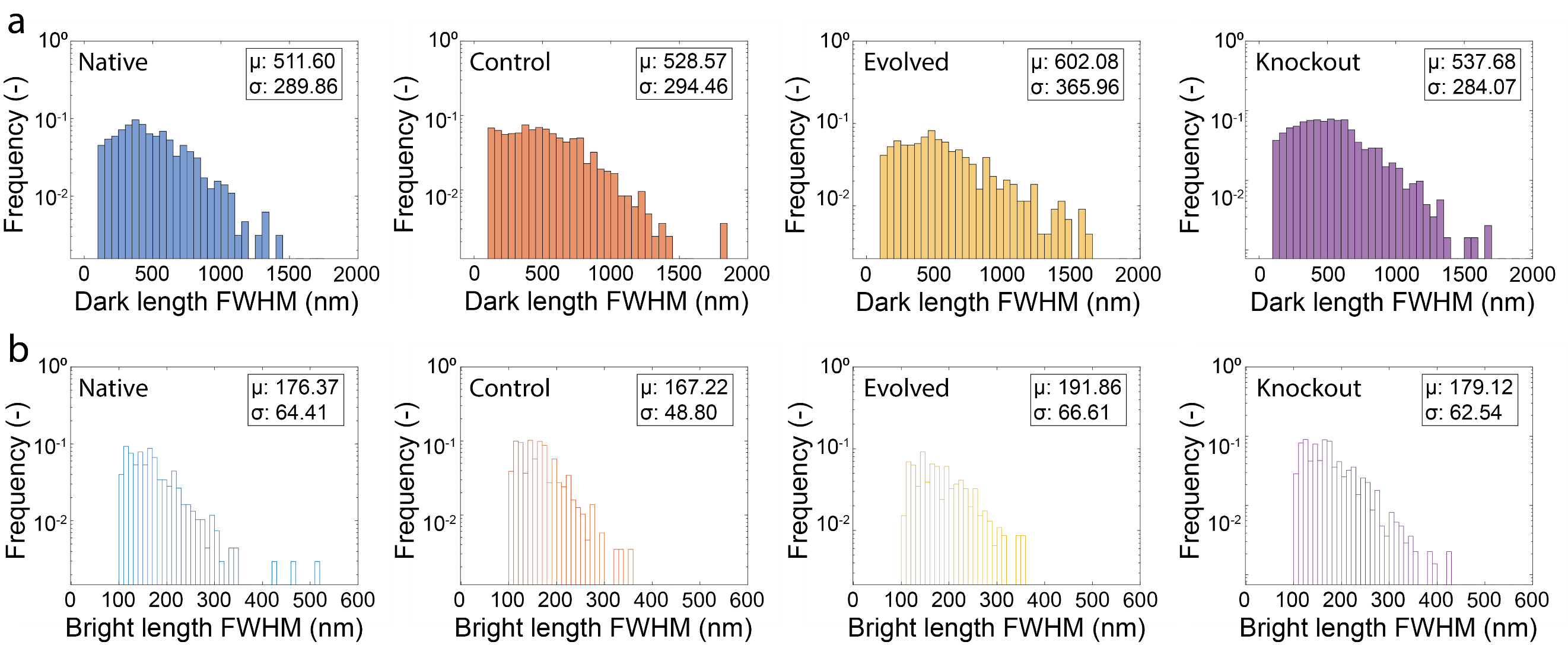


**Figure S2. Data extracted from image analysis of single bacterial cellulose fibers in a microfluidic chamber.** Histograms of the **(a)** dark length (peak spacing at half maximum) and **(b)** bright length (peak full width at half maximum - FWHM) extracted from the line profiles along single bacterial cellulose fibers produced by the different strains. For each sample type, μ is the mean length and σ is the standard deviation of the lengths. Along every fiber, an alternating pattern of bright and dark spots was observed, which were assumed based on the fringed-micellar model of cellulose ultrastructure to correspond to amorphous (disordered) and crystalline domains, respectively. [17].


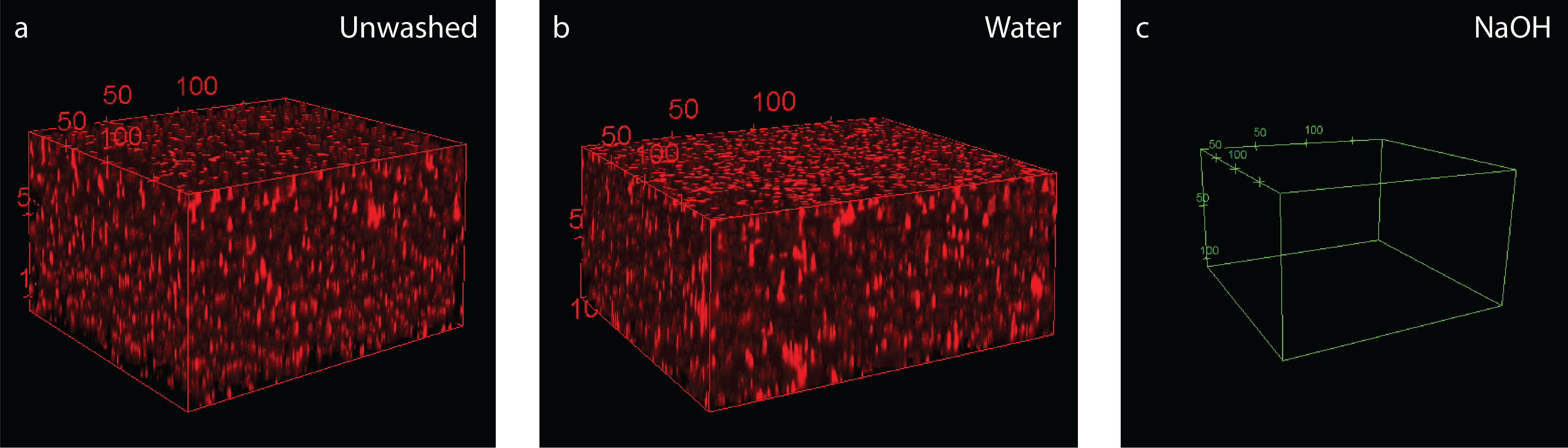


**Figure S3. Effect of washing procedure on the cell content of bacterial cellulose pellicles.** Pellicles were grown from RFP-expressing *K. sucrofermentans* and were either **(a)** left unwashed, **(b)** washed three times with ultra-pure water (UPW), or **(c)** additionally treated with 0.1 M NaOH for 1 h at room temperature and brought back to neutral pH with ultra-pure water. The 3D structures were obtained by stacking confocal microscopy images taken at the top side of the pellicles.


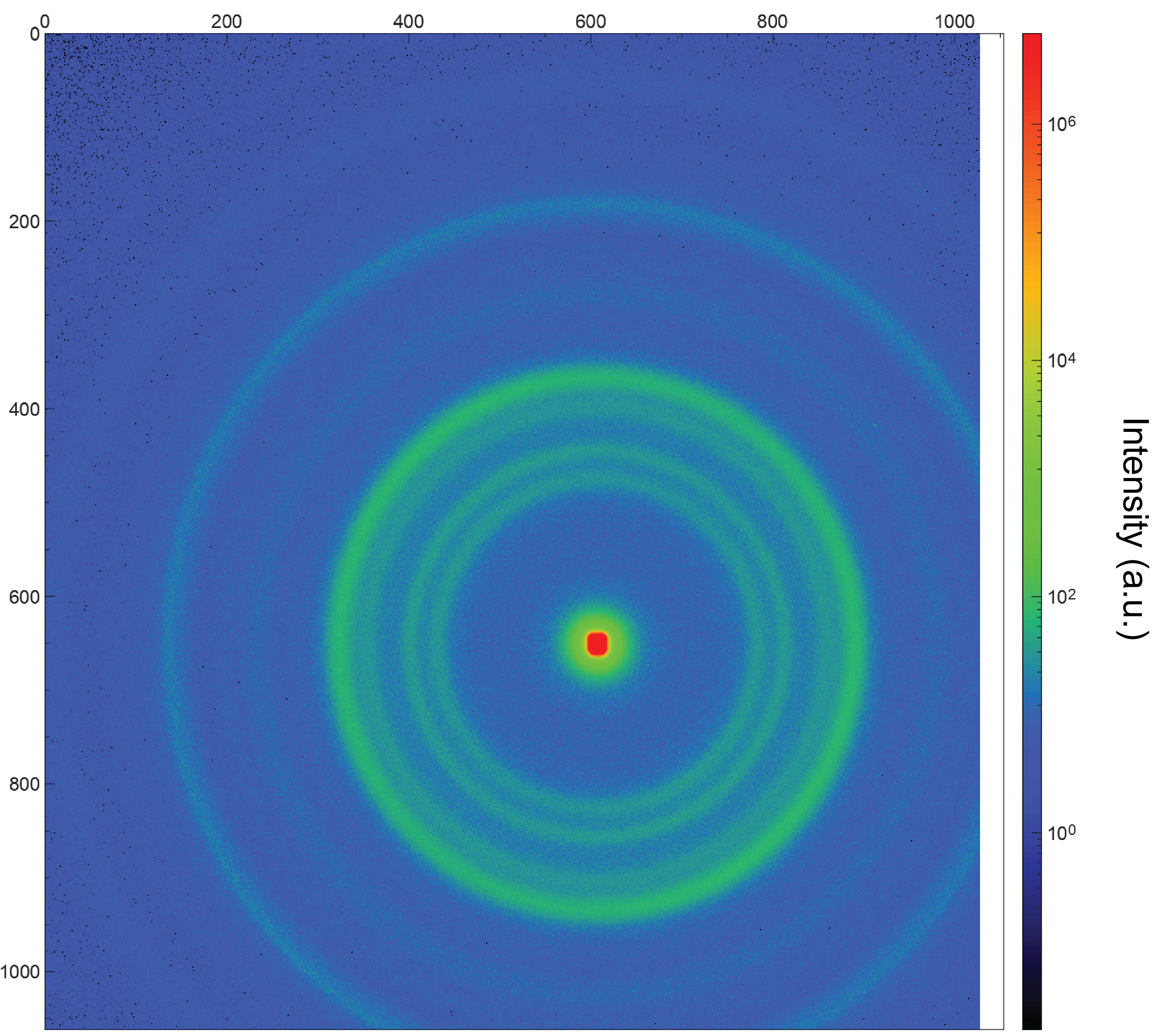


**Figure S4. Representative 2D diffraction data of bacterial cellulose from wide-angle X-ray scattering (WAXS)**. The data is from a freeze-dried bacterial cellulose pellicle produced by the Evolved *K. sucrofermentans* strain and treated with NaOH.


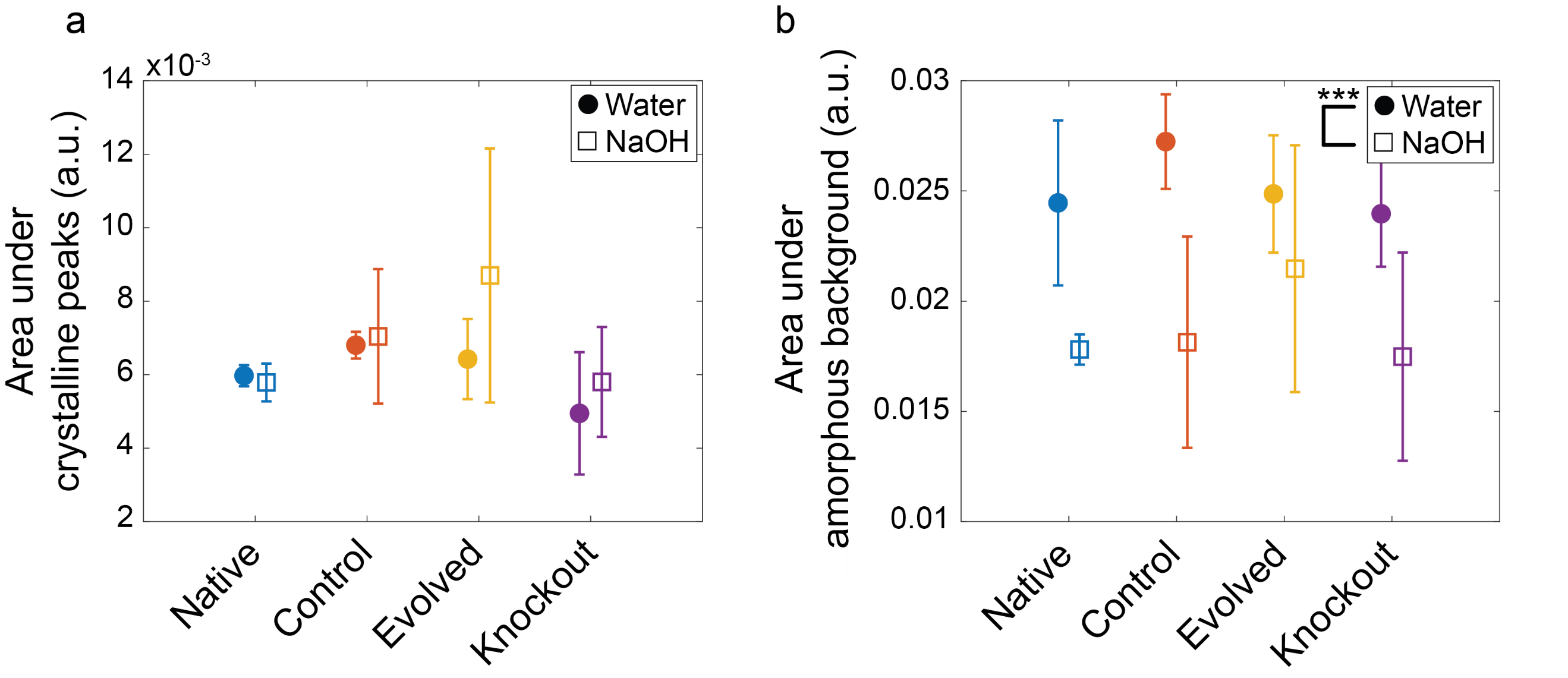


**Figure S5. Effect of strain and washing procedure on the crystallinity and amorphous nature of bacterial cellulose pellicles.** **(a)** Area under the crystalline peaks observed in the wide-angle X-ray scattering (WAXS) diffractograms of samples washed either with water (filled circles) or NaOH (empty squares). The data was obtained by integrating the full diffractogram and subtracting the amorphous background. **(b)** Area under the amorphous background displayed in the WAXS diffractograms. Washing bacterial cellulose pellicles with NaOH significantly reduces the amorphous content. Statistics: ****P* < 0.001, *n_pellicles_* ≥ 3. Error bars represent the standard deviations.


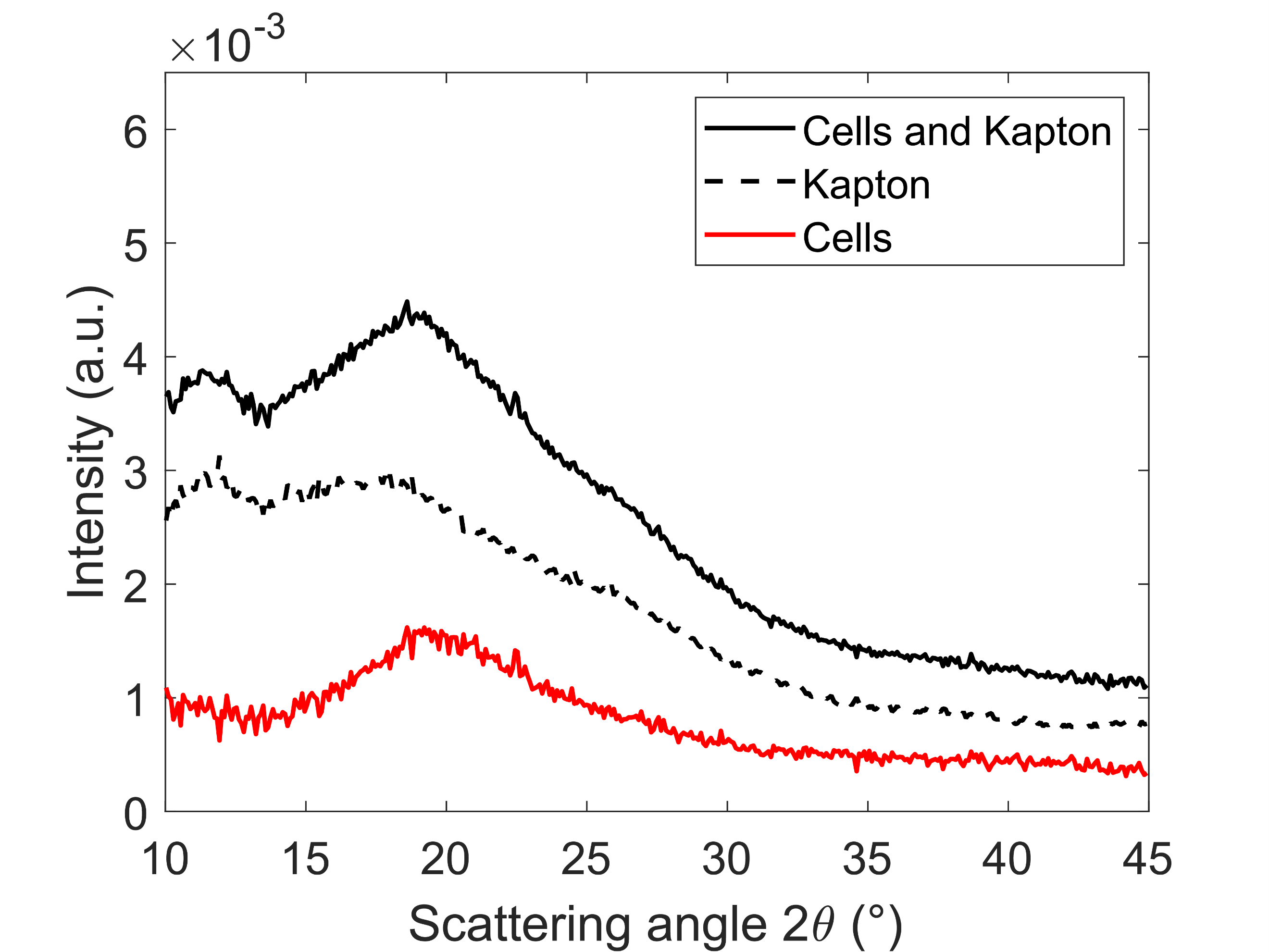


**Figure S6. Wide-angle X-ray scattering (WAXS) diffractogram of *K. sucrofermentans*.** Freeze-dried bacteria were measured in a powder chamber using Kapton films (black curve). An empty powder chamber was measured as a background (dotted black curve). The red curve represents the background-corrected bacterial signal and shows the amorphous nature of cells under those conditions.


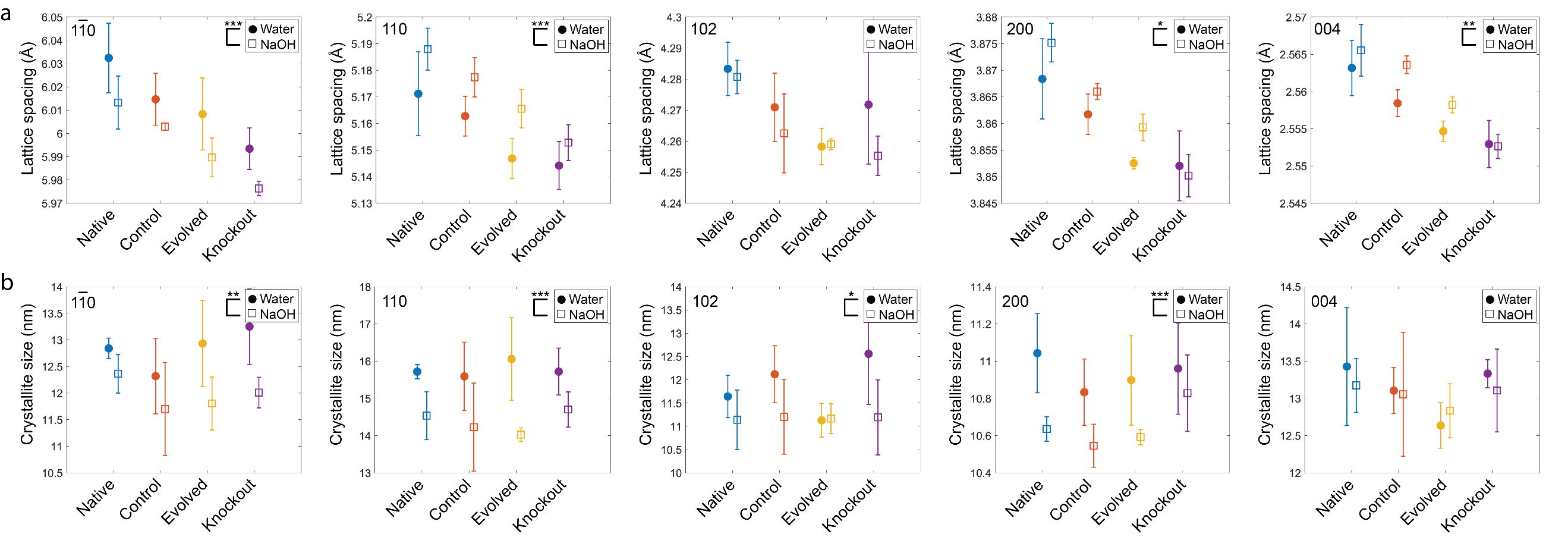


**Figure S7. Crystal structure parameters of freeze-dried bacterial cellulose pellicles produced by the different *K. sucrofermentans* strains.** The data was obtained from the Wide-Angle X-ray Scattering (WAXS) diffractograms. Each panel reports the calculated **(a)** lattice spacing and **(b)** crystallite size for each lattice plane of the cellulose produced by the four different strains. The cellulose was either washed with ultra-pure water (filled circles) or with NaOH (empty squares) before freeze-drying. Statistical differences in the lattice spacing and crystallite size were found between pellicles washed with water or NaOH. Different strains showed a significantly different lattice spacing in all planes (***P* < 0.01, *n_pellicles_* ≥ 3, not shown on graphs). Statistics: **P* < 0.05, ***P* < 0.01, *n_pellicles_* ≥ 3. Error bars represent standard deviations.


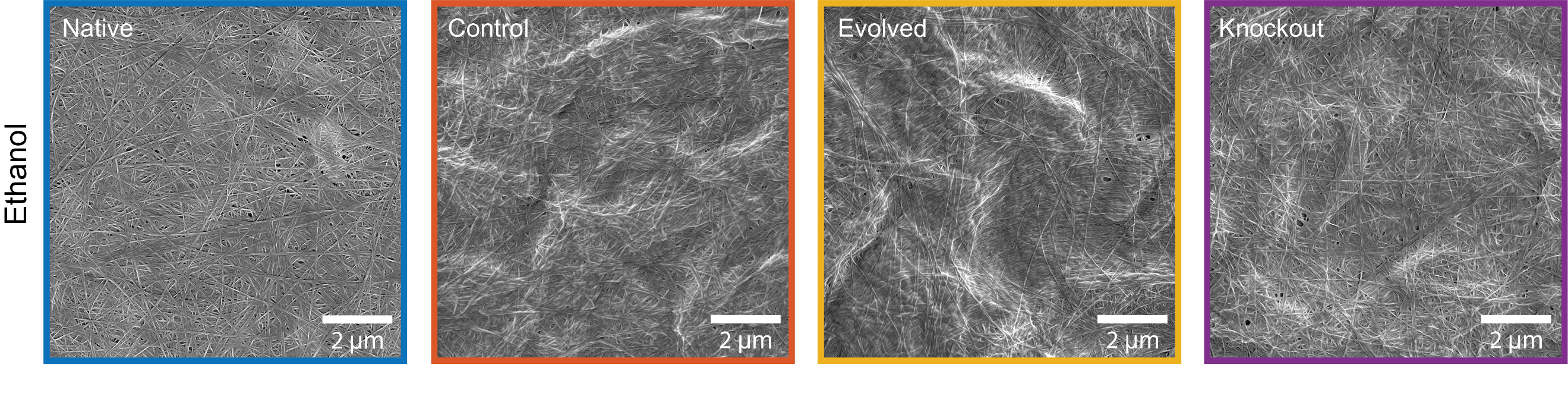


**Figure S8.** **Microstructure of bacterial cellulose pellicles produced by the different *K. sucrofermentans* strains.** Representative SEM images of water-washed bacterial cellulose pellicles, which were then put in aqueous solutions with increasing ethanol content. The fibers collapse into a dense film after that treatment, losing the interfibrillar space.


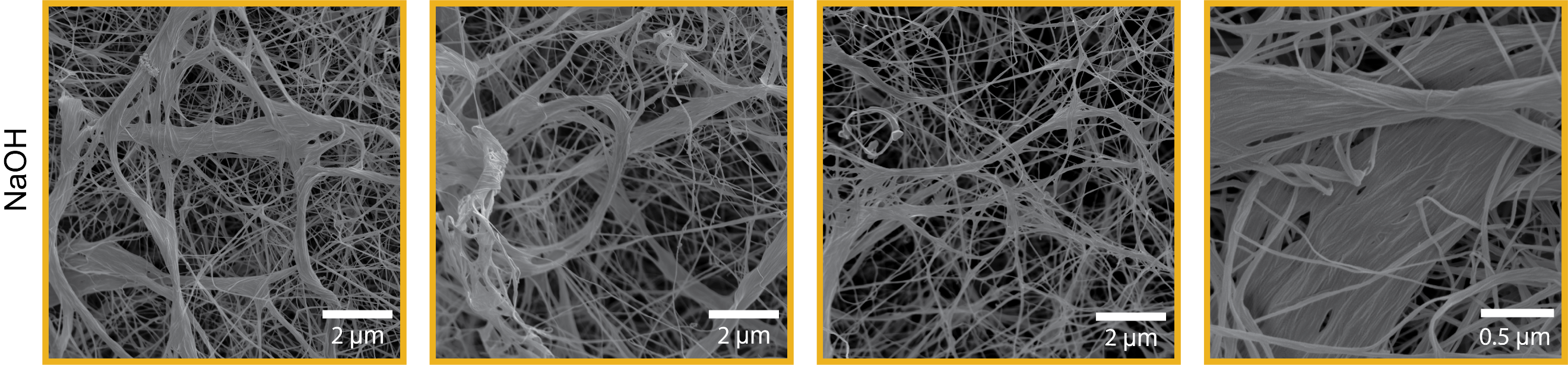


**Figure S9. SEM images of bacterial cellulose pellicles produced by the Evolved *K. sucrofermentans* strain.** Samples were washed with the NaOH treatment.


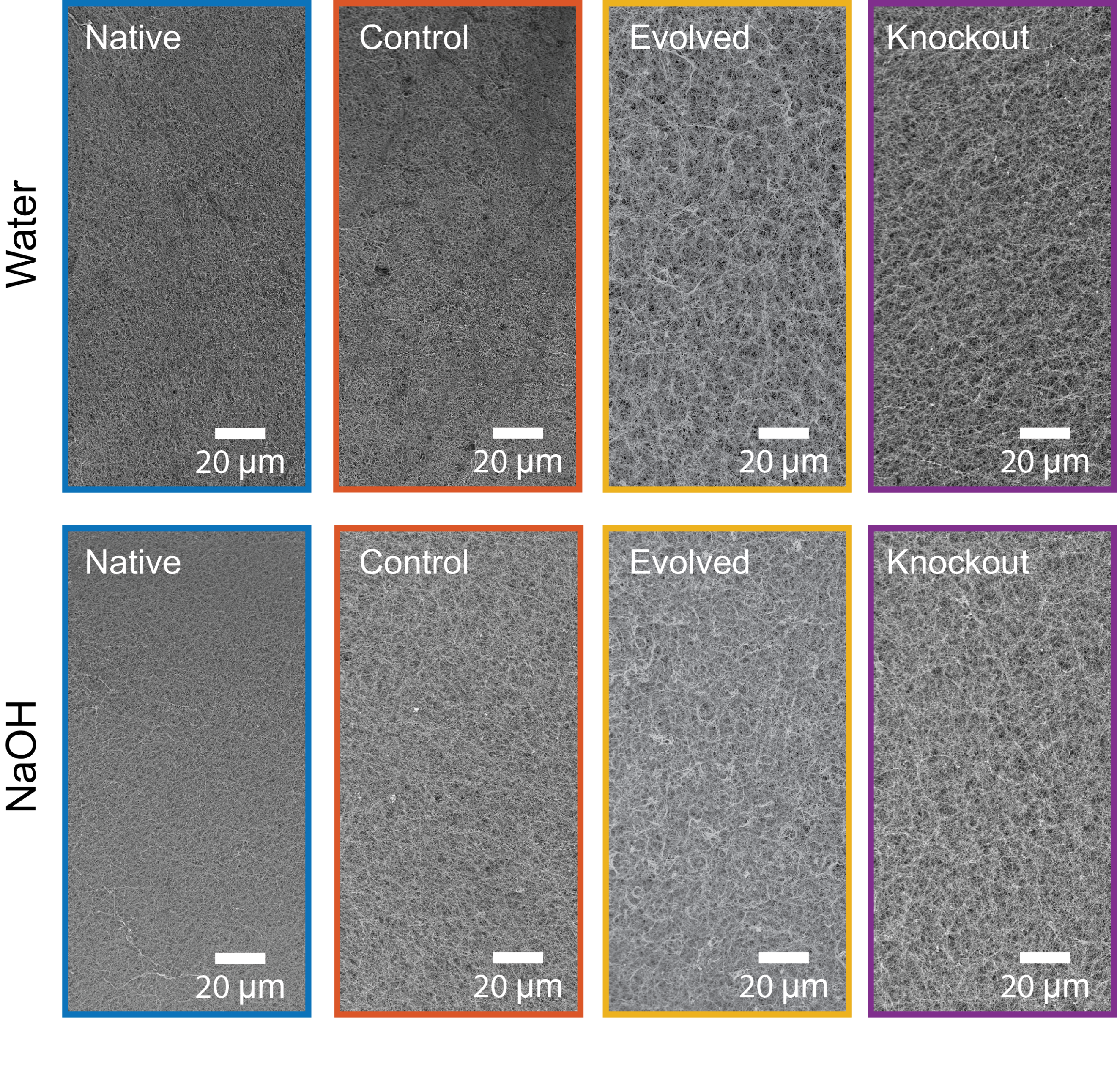


**Figure S10. SEM images of bacterial cellulose pellicles produced by the *K. sucrofermentans* strains at low magnification.**


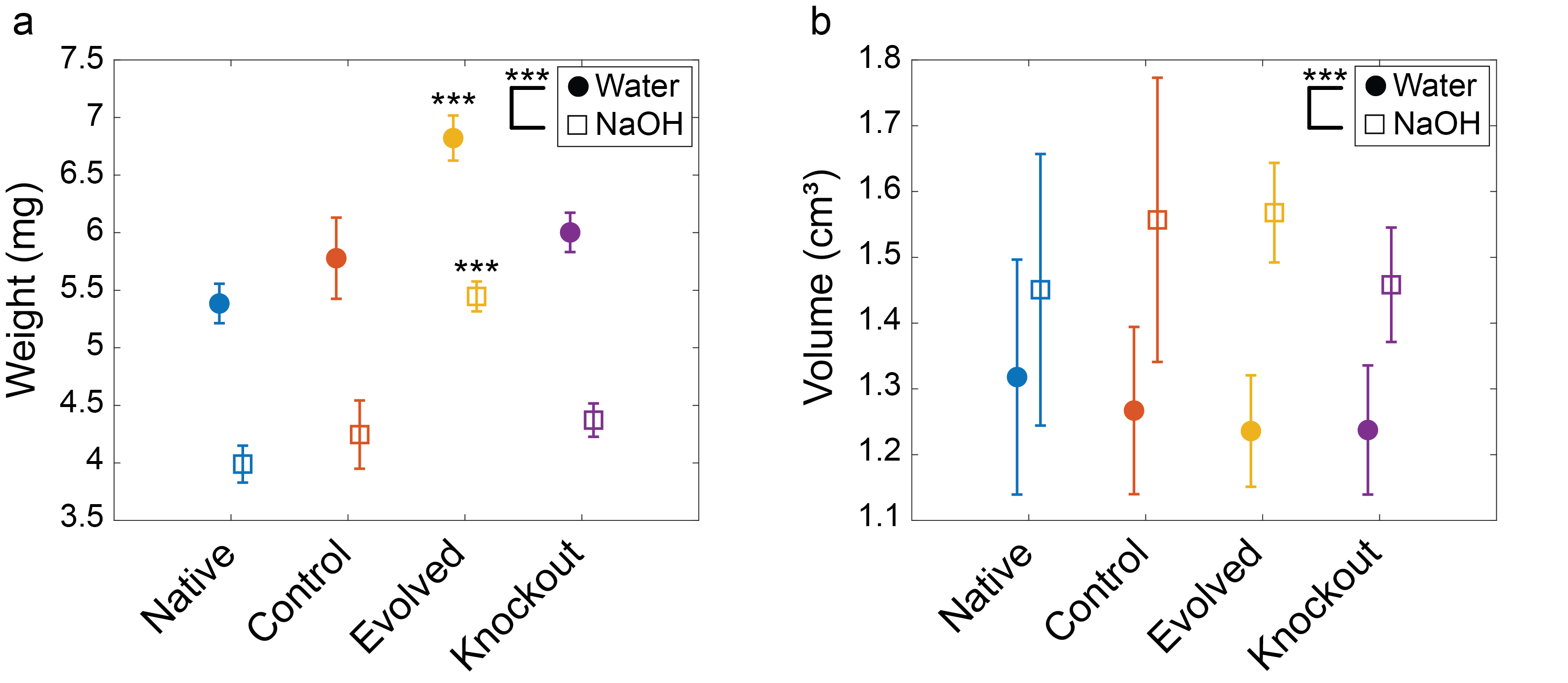


**Figure S11. Weight (a) and volume (b) of bacterial cellulose pellicles made by different *K. sucrofermentans* strains.** The pellicles were either washed with water (filled circles) or with NaOH (empty squares). Washing pellicles with NaOH results both in a statistically significant decrease in dry weight and a statistically significant swelling. Statistics: ****P* < 0.001, *n_pellicles_* = 5. The error bars represent the standard deviation.

**References**

[1] J. G. Hauge, *J. Biol. Chem.* **1964**, *239* (11), 3630, <https://doi.org/https://doi.org/10.1016/S0021-9258(18)91183-X>.

[2] a) R. Hengge, *Nature Reviews Microbiology* **2009**, *7* (4), 263, <https://doi.org/10.1038/nrmicro2109>; b) P. Ross, H. Weinhouse, Y. Aloni, D. Michaeli, P. Weinberger-Ohana, R. Mayer, S. Braun, E. de Vroom, G. A. van der Marel, J. H. van Boom, M. Benziman, *Nature* **1987**, *325* (6101), 279, <https://doi.org/10.1038/325279a0>.

[3] J. L. Wylie, E. A. Worobec, *J. Bacteriol.* **1995**, *177* (11), 3021, <https://doi.org/doi:10.1128/jb.177.11.3021-3026.1995>.

[4] Z. Peng, Z. Lv, J. Liu, Y. Wang, T. Zhang, Y. Xie, S. Jia, B. Xin, C. Zhong, *Carbohydrate Polymers* **2024**, *343*, 122459, <https://doi.org/https://doi.org/10.1016/j.carbpol.2024.122459>.

[5] N. Akasaka, H. Sakoda, R. Hidese, Y. Ishii, S. Fujiwara, *Appl Environ Microbiol* **2013**, *79* (23), 7334, <https://doi.org/10.1128/AEM.02397-13>.

[6] S. H. Kim, H. W. Jang, J. J. Park, D. G. Nam, S. J. Lee, S. H. Yeo, S. Y. Kim, *Antibiotics (Basel)* **2024**, *13* (7), <https://doi.org/10.3390/antibiotics13070626>.

[7] R. R. Singhania, A. K. Patel, M. L. Tsai, C. W. Chen, C. Di Dong, *Bioengineered* **2021**, *12* (1), 6793, <https://doi.org/10.1080/21655979.2021.1968989>.

[8] W. Abidi, L. Torres-Sánchez, A. Siroy, P. V. Krasteva, *Federation of European Microbiological Societies Microbiology Reviews* **2021**, *46* (2), <https://doi.org/10.1093/femsre/fuab051>.

[9] P. M. Fricke, A. Klemm, M. Bott, T. Polen, *Applied and Microbiology and Biotechnology* **2021**, *105* (9), 3423, <https://doi.org/10.1007/s00253-021-11269-z>.

[10] M. Makarem, C. M. Lee, K. Kafle, S. Huang, I. Chae, H. Yang, J. D. Kubicki, S. H. Kim, *Cellulose* **2019**, *26* (1), 35, <https://doi.org/10.1007/s10570-018-2199-z>.

[11] C. M. Lee, N. M. A. Mohamed, H. D. Watts, J. D. Kubicki, S. H. Kim, *The Journal of Physical Chemistry B* **2013**, *117* (22), 6681, <https://doi.org/10.1021/jp402998s>.

[12] J. H. Wiley, R. H. Atalla, *Carbohydr. Res.* **1987**, *160*, 113, <https://doi.org/https://doi.org/10.1016/0008-6215(87)80306-3>.

[13] K. Zhang, A. Feldner, S. Fischer, *Cellulose* **2011**, *18* (4), 995, <https://doi.org/10.1007/s10570-011-9545-8>.

[14] V.-H. Le, M.-C. Caumon, A. Tarantola, A. Randi, P. Robert, J. Mullis, *Anal. Chem.* **2019**, *91* (22), 14359, <https://doi.org/10.1021/acs.analchem.9b02803>.

[15] C. M. Lee, J. D. Kubicki, B. Fan, L. Zhong, M. C. Jarvis, S. H. Kim, *The Journal of Physical Chemistry B* **2015**, *119* (49), 15138, <https://doi.org/10.1021/acs.jpcb.5b08015>.

[16] a) C. A. Schneider, W. S. Rasband, K. W. Eliceiri, *Nature Methods* **2012**, *9* (7), 671, <https://doi.org/10.1038/nmeth.2089>; b) N. Gustafsson, S. Culley, G. Ashdown, D. M. Owen, P. M. Pereira, R. Henriques, *Nature Communications* **2016**, *7* (1), 12471, <https://doi.org/10.1038/ncomms12471>.

[17] M. Babi, A. Palermo, T. Abitbol, A. Fatona, V. Jarvis, A. Nayak, E. Cranston, J. Moran-Mirabal, *ChemRxiv* **2022**, <https://doi.org/10.26434/chemrxiv-2022-0jqng>.
